# Hypoxia inhibits intervertebral disc degeneration by maintaining autophagy circadian rhythm via the HIF1α-PER2-mTOR pathway

**DOI:** 10.1016/j.jare.2025.12.036

**Published:** 2025-12-28

**Authors:** Guang-Cheng Yuan, Qi-Chen Zhang, Yu-Xiang Ge, Heng-Jie Zeng, Tai-Wei Zhang, Wang Ding, Zhi-Rui Dong, Yu-Kai Huang, Jian Dong, Nong Chen, Li-Bo Jiang

**Affiliations:** a Department of Orthopedic Surgery, Zhongshan Hospital, Fudan University, Shanghai 200032, China; b Department of Orhtopedics, Qingpu Branch of Zhongshan Hospital Affiliated to Fudan University, Shanghai 201700, China; c Department of Orthopedic Surgery, Shanghai Xuhui District Central Hospital, Fudan University, Shanghai 200031, China; d Department of Orthopedic Surgery, Minhang Hospital, Fudan University, Shanghai 201100, China; e Department of Orthopedic Surgery, Jinshan Hospital of Fudan University, Shanghai 201508, China

**Keywords:** Circadian rhythm, Autophagy, Hypoxia, Intervertebral disc degeneration, HIF1α

## Abstract

**Introduction:**

Disruption of the circadian rhythm (CR) and autophagy in intervertebral discs contributes to intervertebral disc degeneration (IDD) progression. However, the circadian regulation of autophagy requires further investigation.

**Objectives:**

We observed the expression of circadian proteins and autophagic markers of nucleus pulposus (NP) cells followed a diurnal rhythmic pattern in vivo and in vitro.

**Methods:**

NP tissues were collected from light/dark cycle–shifted rats and IDD patients of varying severity. CR and ECM-related proteins were analyzed by immunohistochemistry and western blotting. Primary rat NP cells were treated with hypoxia or CoCl_2_, followed by western blotting for CR proteins, HIF-1α, and autophagy markers. siRNA knockdown of PER2 or HIF-1α was performed to assess their roles in regulating autophagy, ECM, and CR-associated proteins.

**Results:**

The silencing of clock gene PER2 disrupted the rhythmic expression of autophagic markers, by contrast PER2 overexpression inhibited mTOR pathway and enhanced autophagic levels. Co-IP analysis demonstrated the PER2-mTOR interaction, linking CR and autophagy rhythm. The inflammatory stimulator dampened the CR and autophagy rhythm, however intermittent hypoxia and cobalt chloride (CoCl_2_) re-synchronized the rhythm. Mechanistically, HIF-1α-mediated regulation of PER2 by hypoxia was involved in the re-synchronization, which was further demonstrated by the loss of CR and autophagy rhythm after silencing of PER2 or HIF-1α under hypoxia. Furthermore, the rhythm of oxygen level and HIF-1α was proved in living healthy NP tissue, confirming the hypoxia as a Zeitgeber. CR disruption and autophagy dysfunction led to catabolism of extracellular matrix (ECM), but the hypoxia, CoCl_2_ and autophagic stimulator could promote the rebalance of ECM metabolism.

**Conclusion:**

Our study demonstrates that hypoxia maintains the intrinsic CR and autophagy rhythm through the HIF-1α/PER2/mTOR pathway to prevent IDD.

## Highlights

• PER2 binds mTOR to regulate circadian-autophagy rhythm.
• Hypoxia acts as Zeitgeber via HIF-1α for rhythm resynchronization.
• HIF-1α/PER2/mTOR pathway prevents ECM catabolism in disc degeneration.
• Hypoxia-mimetics restore autophagy rhythm to block matrix degradation.

## Graphical abstract

Hypoxia functions as a physiological Zeitgeber in nucleus pulposus cells, entraining circadian rhythms through HIF-1α stabilization. Under hypoxic conditions, HIF-1α escapes PHD2-mediated degradation and translocates to the nucleus, where it regulates *PER2* expression. PER2, a core circadian component, inhibits mTOR signaling, thereby enhancing autophagic activity in a rhythmic manner. This circadian-autophagy coupling prevents the ECM catabolism, in contrast, disruption of this HIF-1α–PER2–mTOR axis under normoxia or inflammation leads to autophagy dysfunction, ECM degradation, and progressive disc degeneration. Thus, hypoxia maintains disc homeostasis by synchronizing autophagic rhythms.

**Figure:**
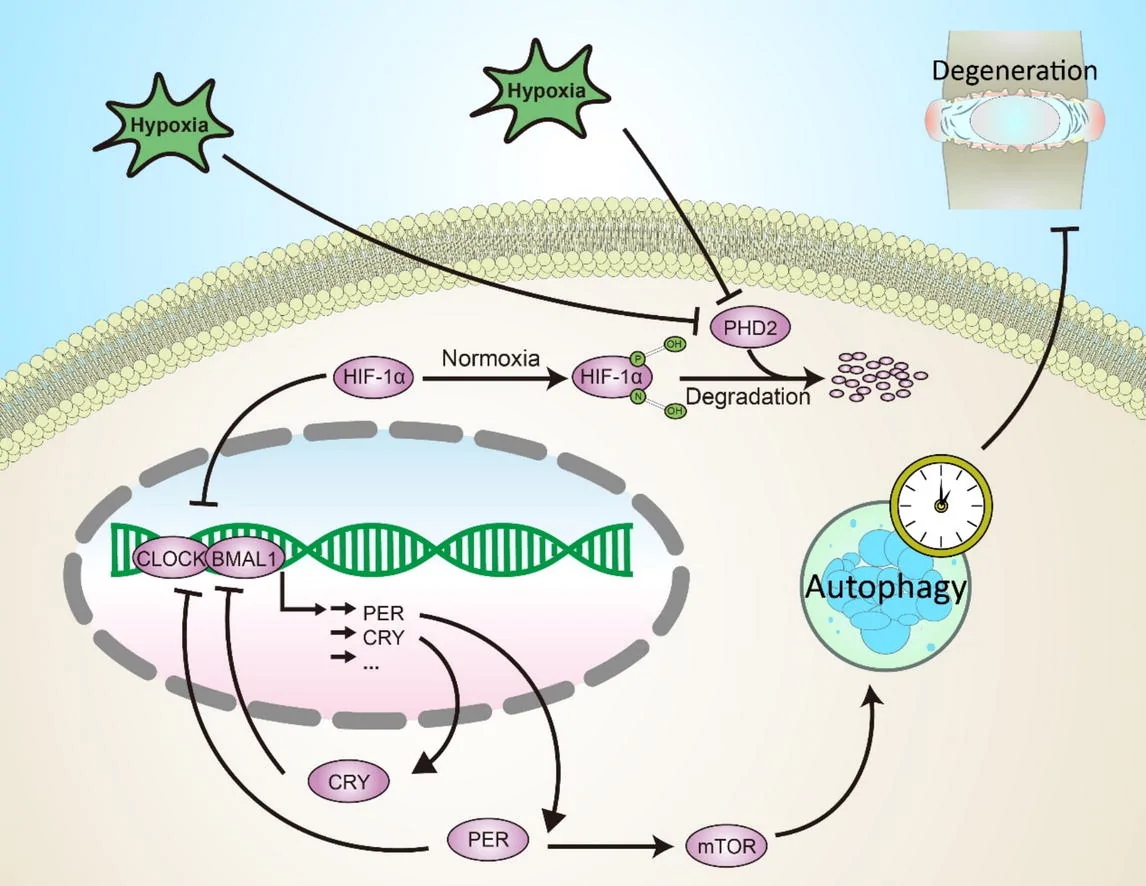

## Introduction

Approximately two-thirds of adults suffer from lower back pain. The most common causes are age-related degeneration of the lumbar intervertebral disc (IVD) and disc herniation [1]. Each IVD is composed of a cartilage endplate, nucleus pulposus (NP), and annulus fibrosus [2]. These three components are involved in intervertebral disc degeneration (IDD) [3,4]. Diurnal changes in mechanical load lead to diurnal changes in osmotic pressure, and mechanical loading together with other microenvironmental factors, provide stimuli for NP cells [[5], [6], [7]]. Recently, a self-regulating circadian rhythm (CR) was discovered in IVDs, and disruption of the CR in NP cells could lead to IDD [8].

The CR is a time-measuring mechanism that exists in most photosensitive organisms. The central nervous system interacts with surrounding tissues in a 24 h oscillation cycle to control nutrient metabolism, energy balance, redox state, and body behavior, and to maintain homeostasis through complex pathways [9]. Mammalian cells also possess an autonomous molecular CR, which is produced by two chained transcription/translation feedback loops (TTFL), which generate a powerful 24 h rhythm of gene expression [10]. The core TTFL is driven by four complete clock proteins, including two activating factors, CLOCK (circadian locomotor output cycles kaput) and BMAL1 (Brain and Muscle ARNT-Like 1), and two repressors, Per (Period) and Cry (Cryptochrome). In addition, kinases (CKIα, CKIδ, and CKIɛ) and phosphatases (PP1, PP5) regulate phosphorylation, thereby regulating the positioning and stability of these integrated clock proteins [10]. The NP has recently been found to possess a CR; this is a key factor affecting the degeneration of the NP [11]. However, it is unclear how to maintain CR stability in NP cells, because the IVD does not have a blood supply, and NP cells reside in an ischemic and hypoxic microenvironment without connection to central rhythm regulators [12].

The oxygen concentration of IVDs in dogs was measured via the electrode method, and oxygen tension in the NP was found to be approximately 0.53–1.06 kPa (4–8 mmHg), which was significantly lower than that in the outer annulus fibrosus (7.5 kPa) and other organs [13]. The hypoxic environment maintains NP homeostasis and alleviates the compression-induced apoptosis of NP cells [14,15], therefore, a hypoxic environment is beneficial for NP cells under ischemic and compressive stress conditions. This hypoxic microenvironment is regulated by exercise and synchronizes with changes in the oxygen content of blood [16,17]. Recent studies have revealed a CR in the oxygen content of blood flow and multiple organs, and have shown that oxygen can act as an independent regulator of the peripheral CR [18]. Hypoxia can lead to misalignment of the circadian clock in the kidneys and lungs [19]. However, due to the hypoxic environment of NP cells, it would be of interest to determine the role of oxygen in regulating changes in the CR in IVDs.

Autophagy is a basic cellular process in eukaryotes that is essential for individual cells or organs to respond to, and adapt to, changing environments. In this process, cells digest themselves to produce life-sustaining substance [20]. 5-monophosphate-activated protein kinase (AMPK) directly phosphorylates Ulk1 to promote autophagy [21]. Additionally, the activation of AMPK induces Raptor phosphorylation, which inhibits the inhibitory effect of mammalian target of rapamycin complex 1 (mTORC1) on Ulk1 autophagy complexes, inducing autophagy [22]. In NP cells, autophagy has been shown to maintain cell homeostasis, inhibit apoptosis, and prevent aging [23]. However, whether autophagy is rhythmic in NP cells remains unclear.

Recently, evidence has emerged of an interaction between CR and autophagy. Many genes are involved in the autophagy pathway, including autophagy related 14 (*Atg14*)*, Ulk1, Gabarapl1, LC3B*, and *BCL2/Bnip3* [24]. As an upstream element of autophagy, PER2 specifically inhibits the activity of the mTORC1 complex in HEK293T cells by binding to each protein of the complex. Because PER2 is a core element of the CR, its periodic expression periodically activates and inhibits the mTORC1 complex [25]. Knockdown of PER2 reduces the level of autophagy while maintaining the core CR oscillations [26]. Whether this regulation occurs in NP cells and plays a potential role in IDD requires further study. In the present study, we established a connection between autophagy and CR and elucidated the specific role of hypoxia in regulating CR stability in NP cells.

## Materials and Methods

### Patients and clinical specimens

The acquisition of patient specimens and experimental procedures in this study were approved by the Ethics Committee of Zhongshan Hospital Affiliated to Fudan University. This study was conducted in full accordance with the rules and regulations of the Ethics Committee of Zhongshan Hospital Affiliated to Fudan University. From February 2019 to November 2020, NP tissue specimens were collected from 10 patients with lumbar disc herniation or spinal fractures who underwent intervertebral discectomy. The specimens were divided into two groups: one was immediately placed in 4 % paraformaldehyde and used to generate paraffin-embedded sections for immunohistochemistry (IHC); the other was immediately placed in high-glucose Dulbecco's Modified Eagle Medium (DMEM) containing 15 % fetal bovine serum (FBS) for primary NP cell isolation. Patient information is listed in Supplementary Table 1.

### Laboratory animals

The experimental design was approved by the Animal Protection Committee of Zhongshan Hospital Affiliated to Fudan University. The handling and use of animals followed the “Guidelines for the Care and Use of Laboratory Animals” of the National Department of Health. For this experiment, 50 4-week-old specific pathogen-free (SPF) male Sprague-Dawley (SD) rats and 12 2.5 kg male New Zealand rabbits were purchased from Shanghai Jiesjie Experimental Animal Center and raised in the Experimental Research Center of Zhongshan Hospital Affiliated to Fudan University. Animals were anesthetized using isoflurane (2–3 % in 100 % oxygen) via inhalation until loss of consciousness. Euthanasia was performed using CO_2_ inhalation, followed by cervical dislocation to confirm death.

### Isolation and culture of NP cells

To obtain human NP cells, NP tissue isolated from patients’ spines was soaked in high-glucose Dulbecco’s Modified Eagle’s Medium (DMEM; Gibco, Grand Island, NY, USA, 11995065) containing 15 % FBS and cut into small pieces. To obtain rat NP Cells, 4-week-old SD rats were sacrificed, and NP tissue was isolated using micro-instruments. Collagenase P (Roche, Basel, Switzerland, 11249002001) and TrypLE™ Express (Gibco, 12605028) were used to digest the tissue at 37 °C for 4 h and 30 min. NP cells were then seeded in DMEM containing 10 % FBS (Gibco, 10099-141C) and antibiotics (1 % penicillin/streptomycin), and cultured at 37 °C in a humidified incubator with 95 % air and 5 % CO_2_ (passage 0). When the cells reached 80–90 % confluence, they were detached with 1 × TrypLE Express Enzyme and subcultured in culture flasks. Passage 2 NP cells were used in all the experiments. The NP cell phenotype was confirmed using collagen II immunofluorescence, Alcian blue staining, and toluidine blue staining. Each experiment was repeated three times.

### Transfection

When NP cells reached 80–90 % confluence, they were transfected with target siRNA (listed in Supplementary Table 2) or non-targeting control siRNA (both from RiboBio, Guangzhou, China) at a final concentration of 50 nM with Opti-MEM medium (Gibco, A41248) using Lipofectamine 3000 reagent (Invitrogen, Carlsbad, CA, USA, L3000) according to the manufacturer’s instructions.

When NP cells reached 80–90 % confluence, they were transfected with pLVX-EF1α-PER2-mCherry plasmid at a final concentration of 5 μg/μL with Opti-MEM medium (Gibco, A41248) using Lipofectamine 3000 reagent (Invitrogen, Carlsbad, CA, USA, L3000) according to the manufacturer’s instructions. Fluorescence intensity was used to determine the transfection efficiency. After 72 h, the cells were harvested and the protein was extracted.

### Co-Immunoprecipitation

Cells cultured in a 10 cm dish were used for Co-IP experiments. Protease inhibitors were added at a ratio of 1:100 in the Co-IP lysis solution, and phosphatase inhibitors were added at a ratio of 1:50. Cells were then placed on ice, washed three times with phosphate-buffered saline (PBS), and 1 mL of Co-IP lysis solution was added to each 10 cm dish. The cells were scraped using a cell scraper and the lysate was collected. The lysate was placed in a homogenizer (Jingxin Industrial Development Co., Ltd, Shanghai, China) and vibrated at 60 Hz and 4 °C for 30 min. Samples were centrifuged at 12000 rpm for 5 min, and the precipitate was discarded. Then, 100 μL of supernatant was harvested as the input. Following a wash with fresh Co-IP lysis buffer, protein A/G immunomagnetic beads and 2 μL of the corresponding antibody were added to the remaining 900 μL of supernatant. The mixture was then incubated under slow rotation overnight at 4 °C. The solution was placed on a magnetic stand. After the magnetic beads were collected, 100 μL of the supernatant was harvested and used as the supernatant. The magnetic beads were washed three times with fresh Co-IP lysis solution, and 50 μL 1 × protein loading buffer was used to resuspend the magnetic beads. These were then placed in a metal bath at 95 °C for 10 min in a PCR machine, labeled as IP.

### Total RNA isolation and reverse Transcription-Polymerase chain Reaction analysis

Total RNA was extracted from NP tissue and cells using TRIzol reagent (Invitrogen, 15596026), and reverse transcribed to form cDNA with PrimeScript RT Master Mix (Takara, Tokyo, Japan, RR036A), according to the manufacturer’s protocol. Relative mRNA levels were determined using a TB Green™ Premix Ex Taq™ II (Takara, RR820) with an ABI QuantStudio5 sequence detector (Applied Biosystems, Waltham, Massachusetts, USA). RT-PCR data were analyzed using the ΔΔCt method [11]. *Gapdh* or *Actb* mRNA levels were used as internal controls. Primers used for RT-PCR analysis are shown in Supplementary Table 3.

### Protein extraction and western blotting

Following treatment, cell culture plates were placed on ice and NP cells were rinsed with ice-cold PBS and then lysed in ice-cold radioimmunoprecipitation assay buffer containing Halt Protease and Phosphatase Inhibitor Cocktail (Thermo Fisher Scientific, MA, USA; #78444). Protein was extracted from rat NP tissue with TRIzol reagent (Invitrogen) according to the manufacturer’s protocol. Total proteins were electrophoretically separated on an 8–12 % polyacrylamide gel and transferred to an Immobilon-PSQ polyvinylidene difluoride membrane (Millipore, Billerica, MA, USA, ISEQ00010), blocked with QuickBlock™ Primary Antibody Dilution Buffer for Western Blot (Beyotime, Shanghai, China) for 1  h, and then incubated overnight at 4 °C with primary antibodies (antibodies used in this study and their dilutions were listed in Supplementary Table 4). Membranes were incubated with horseradish peroxidase (HRP)-conjugated secondary antibodies at room temperature for 1 h after washing three times with Tris-buffered saline with Tween (TBST). Immobilon Western Chemiluminescent HRP Substrate (Millipore, WBKLS0100) was used for enhanced chemiluminescence and signal intensity was analyzed with ImageJ software (National Institutes of Health, Bethesda, MD, USA).

### Bioinformation analysis

We browsed our previous RNA sequencing data [11]. Differentially expressed genes were identified as those with a false discovery rate < 0.05, fold change > 2.0, and Fragments Per Kilobase per Million (FPKM) ≥ 0.1 in at least one group. ClusterProfiler was used for KEGG terms enrichment analysis [27]. Heat maps and bubble plots were generated using the pheatmap package [28].

### Intervertebral disc oxygen concentration measurement

New Zealand rabbits (male, 2.5 kg) were anesthetized with 20–60 mg/kg pentobarbital and placed in the right decubitus position. The hair on the left-side of the back was shaved, and an incision was made along a straight line parallel to the spine through the iliac crest, cutting through the anterolateral muscle gap, and exposing the L2/3, layer by layer from the retroperitoneum. To the L5/6 IVD, gauze was used to carefully expose the anterior and outer sides of the L3/4 and L5/6 IVDs after being positioned by X-ray. A 16G puncture needle was used to puncture the L3/4 and L5/6 IVDs to a depth of 5 mm, the needle tip was rotated 180°, paused for 30 s, and then removed (Fig. S8A–D). The wound was sutured layer-by-layer. Animals received 500,000 units of penicillin injected intramuscularly for 7 days following the procedure.

One month after the model was established, the IVD was observed with a thickness of 1 mm using the T2_tse_fs sequence under a SIEMENS skyra 3.0 T MR instrument to determine its degeneration. For New Zealand rabbits in which the model was successful, the IVDs were exposed along the old approach, and a calibrated PM-PSt7 micro-oxygen probe (PreSens, Regensburg, Germany) was used to puncture the modeled IVDs and adjacent healthy vertebral discs. The puncture site was confirmed by X-ray, and the sensor probe was removed to determine the oxygen content in the IVD (Fig. S8E–I). During the measurement, the venous channel was retained, and anesthetics were added.

### Histological evaluation and immunohistochemistry

Human and rat tissues were fixed with 4 % formaldehyde, decalcified with 10 % ethylenediaminetetraacetic acid solution, and embedded in paraffin for serial sectioning. After deparaffinization, the sections were stained with hematoxylin and eosin (HE) or safranin-O. Other sections for IHC were incubated with 3 % H_2_O_2_ for 10 min and 0.1 % trypsin for 20 min, followed by incubation with primary antibody (Supplementary Table 5) at 4 °C overnight. Finally, the sections were incubated at 37 °C for 2 h with HRP-conjugated secondary antibodies and counterstained with hematoxylin. Binding of the secondary antibody was visualized through 3,3-diaminobenzidine tetrahydrochloride solution (Beyotime, Shanghai, China). At least three sections from each human NP tissue specimen were used to evaluate protein expression.

### CR disorder model

The CR disorder model was established following a previous protocol [11]. Briefly, 24 3-week-old male SD rats were randomly divided into control and shifted groups (CR disorder models). All animals were initially fed under 12:12 LD cycles for 1 week. For the shifted group, the rats were fed under 24:24 LD cycles for 3 weeks and then transferred into conventional 12:12 LD cycles for 1 week to prevent adaptation. The control group was fed under the constant 12:12 LD cycles. Rats were sacrificed 12 months after the procedure, and lumbar IVDs were collected for IHC analysis.

### CR analysis

CircWave v1.4 (https://www.clocktool.org/clock-modules/clock-tools/item/circ-wave.html) was used to fit the CRs of protein/RNA expression at different time points, and the control. A suitable fitting curve was identified for gene expression for the α value. When an oscillating curve was obtained at α < 0.05, the gene was considered to present rhythmic expression. Otherwise, the gene was not considered to possess rhythmic expression, and a horizontal straight line was obtained at α = 0.05.

## Results

### Autophagic activity of the NP possesses a rhythm in vitro and in vivo

Identification of rat and human NP cells was depicted in Fig. S1A–F and Fig. S1G–L respectively. Protein expression levels were evaluated 48 h after synchronization with dexamethasone (Dex) (Fig. 1A and B, Fig. S2A). In unsynchronized NP cells, the expression of BMAL1 protein was found to follow a circadian rhythm (CR). Upon Dex synchronization, CR-related proteins BMAL1 and CLOCK, as well as autophagy-associated proteins P62 and microtubule-associated protein 1A/1B-light chain 3 (LC3-II), also conformed to a CR pattern. This observation suggests that rat NP cells exhibit a circadian rhythm, supported by biomaterial strategies that activate intrinsic circadian regulation through BMAL1-CLOCK interactions and PI3K-AKT signaling, which collectively enhance extracellular matrix synthesis and mitigate intervertebral disc degeneration (IDD) [29]. Furthermore, the autophagic activity of NP cells was found to possess rhythmicity. MetaCycle analysis (Fig. S2B) confirmed that Dex treatment reinstated the rhythmic expression of several core clock and autophagy-related proteins. BMAL1 under Dex conditions exhibits a circadian rhythm with a period of 24 h, peak phase at approximately ZT8 and relative amplitude of 0.10; LC3-II under Dex shows a period of 24 h, peak phase at ZT24 and relative amplitude of 0.17; P62 under Dex has a period of 24 h, peak phase at ZT24 and relative amplitude of 0.13; CLOCK under Dex displays a period of 24 h, peak phase at ZT24 and relative amplitude of 0.15. Human NP cells were synchronized with dexamethasone (Dex), and alterations in protein expression were assessed 24 h post-synchronization (Fig. 1C; Fig. S2B). Prior to synchronization, circadian rhythm (CR) and autophagy-related proteins in human NP cells exhibited only a modest rhythm. However, following synchronization, the CR was notably amplified, as evidenced by a marked increase in the amplitude of rhythmic expression of both CR and autophagy-related proteins. This finding aligns with previous studies suggesting that melatonin, a known CR modulator, enhances autophagy and mitophagy in human NP cells via PGC-1α-dependent pathways, thereby restoring mitochondrial homeostasis and counteracting degenerative processes [30]. BMAL1 exhibited a distinct 24-hour oscillation following Dex treatment, with a peak at Zeitgeber Time (ZT) 16, in stark contrast to the nearly flat expression observed under control conditions. LC3-II similarly demonstrated restored rhythmicity under Dex, peaking at ZT 8, and showing synchronized oscillation in relation to BMAL1. Subsequently, caudal intervertebral disc (IVD) NP tissues from rats maintained under a 12:12 light/dark (LD) cycle were harvested to assess the rhythmic expression of circadian rhythm (CR) and autophagy-related proteins. In vivo, the expression of CR-associated proteins BMAL1 and PER2, along with autophagy-related genes P62 and LC3-II, exhibited rhythmic patterns, as did the phosphorylation status of the mammalian target of rapamycin (mTOR) (Fig. 1D). A phase difference of approximately 12 h was observed between the inhibitory phase and the increase in LC3-II, suggesting that the circadian rhythm in NP cells can be induced in vitro by pharmacological agents and is also present in vivo. This observation is consistent with previous reports indicating that disruption of the CR accelerates the progression of intervertebral disc degeneration (IDD) through mechanisms such as sympathetic nervous system dysregulation, which compromises disc homeostasis and exacerbates inflammatory responses [31]. MetaCycle (Fig. S2E) analysis revealed distinct circadian characteristics for several key proteins. BMAL1 exhibited a peak expression at Zeitgeber Time (ZT) 16 with an amplitude of 0.055, showing a phase advance of approximately 1 h relative to phosphorylated mTOR. PER2 peaked at ZT 21 with an amplitude of 0.132, corresponding to a phase delay of about 4 h. LC3-II reached its maximum at ZT 8 with an amplitude of 0.096, representing a phase advance of roughly 8 h, and showed a significant rhythmicity (q = 0.001). Phosphorylated mTOR (p-mTOR) displayed a peak at ZT 16 with an amplitude of 0.189 and served as the phase reference (q < 0.001). Finally, p62 peaked at ZT 9 with an amplitude of 0.227, also exhibiting a phase advance of about 8 h. Although autophagy and circadian clock-related proteins present rhythmic oscillations in NP cells following Dex addition, it is still unknown whether circadian oscillations in autophagy are regulated by CR-related proteins. KL001, a stabilizer of Cry protein, can inhibit the degradation of Cry, and thus inhibit the 24 h CR oscillations, consistent with observations that CRY modulation alters clock gene feedback loops and influences downstream cellular processes like autophagy in degenerative models [32]. Initially, it was demonstrated that 2.5 and 5 μM KL001 significantly reduced the mRNA expression of PER2 and Arntl (Fig. S3A). Subsequently, it was observed that 5 μM KL001 abolished the oscillatory expression of both circadian clock-related and autophagy-related proteins in rat NP cells 48 h after synchronization, thereby suggesting a regulatory relationship between the circadian rhythm (CR) and autophagy rhythm (Fig. S3B–D).

**Fig. 1:**
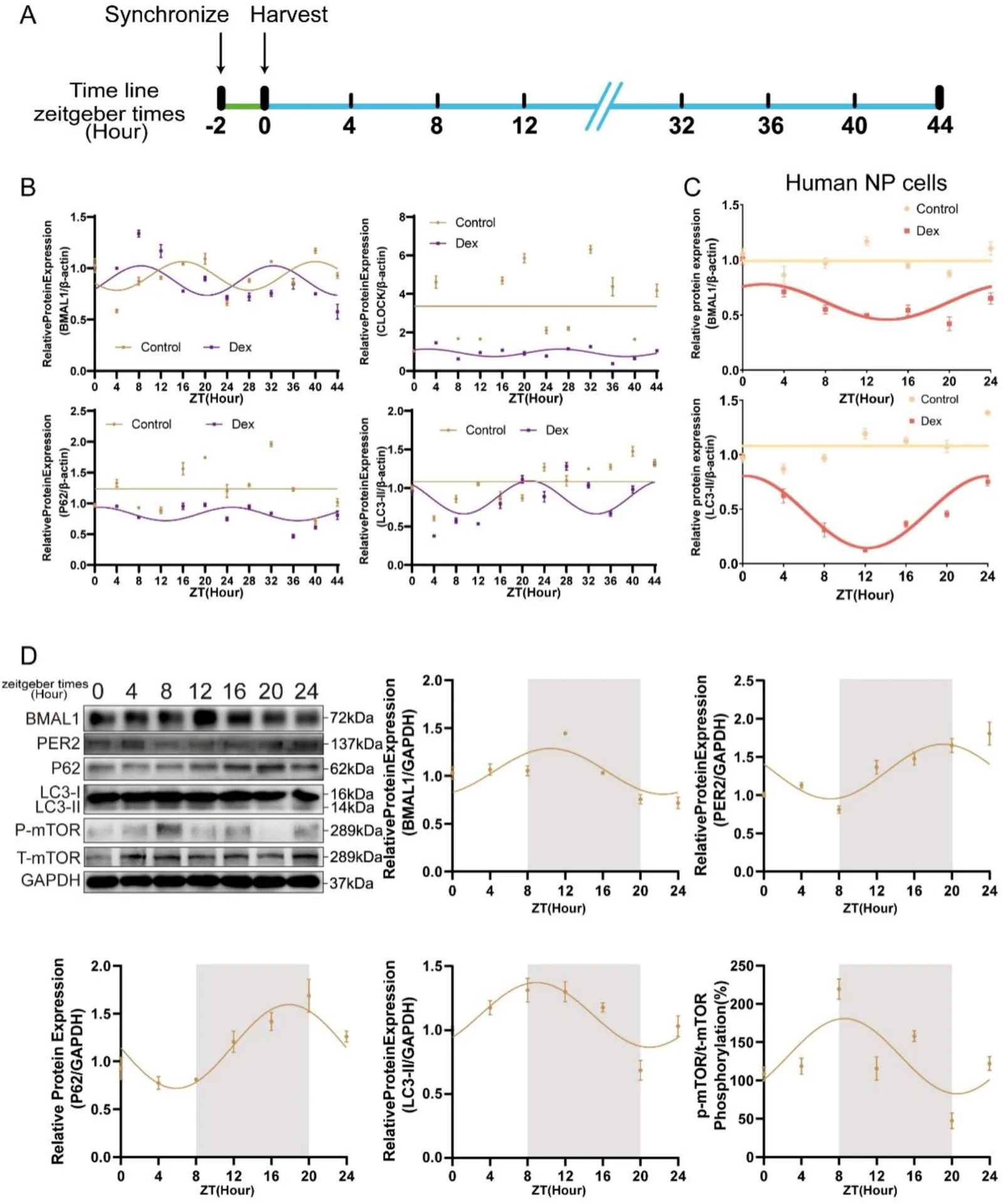
Rhythmic expression of clock and autophagy related proteins. A. Timeline of synchronization of rat NP cells with dexamethasone. B. Rhythmic expression of BMAL1, CLOCK, P62, and LC3-II protein in dexamethasone-synchronized rat NP cells, n = 3. Data are superimposed with corresponding sine waves fitted through CircWave analyses. C. Rhythmic expression of BMAL1 and LC3-II protein in dexamethasone-synchronized human NP cells, n = 3. Data are superimposed with corresponding sine waves fitted through CircWave analyses. D. Rhythmic expression of BMAL1, PER2, P62, and LC3-II protein and rhythmic phosphorylation of mTOR in rat NP tissues, n = 3. The gray panels indicate the night (dark) periods. Data are superimposed with corresponding sine waves fitted through CircWave analyses.

### The circadian rhythm regulates the autophagy rhythm of rat NP cells through the PER2-mTOR pathway

The mechanism underlying the relationship between circadian rhythm (CR) and autophagy was further investigated. A prior study demonstrated that PER2 interacts with and inhibits mTORC1 in HEK293T cells, suggesting a potential regulatory link between the circadian clock and autophagy through the modulation of mTORC1 activity [25]. We hypothesized that PER2 plays a pivotal role in linking circadian rhythm (CR) and autophagy. To test this, PER2 was knocked down using siPER2 for 2 days, and NP cells were subsequently synchronized with Dex. At 24 h post-synchronization, a reduction in the levels of both BMAL1 and PER2 was observed in the PER2-knockdown group (Fig. S4A). This resulted in the loss of CLOCK and BMAL1 rhythmicity, and a significant decrease in the amplitude of LC3-II expression (Fig. 2A; Fig. S4D–F). Furthermore, the levels of autophagic proteins ATG7, ATG5, and LC3-II were significantly diminished, while P62 levels were elevated following PER2 silencing (Fig. S4C). These findings align with previous observations that PER2 deficiency disrupts autophagic processes through the enhancement of mTORC1 signaling in circadian-regulated cellular activities [33]. Upon overexpression of PER2, the phosphorylation levels of mTOR, S6, and 4E-BP1 in NP cells were significantly reduced (Fig. 2C). In contrast, following PER2 knockdown, the phosphorylation levels of mTOR and S6 were notably elevated (Fig. S4B). Additionally, the expression of autophagic proteins ATG7 and LC3-II was significantly enhanced upon PER2 overexpression (Fig. 2B and Fig. S2F).

**Fig. 2:**
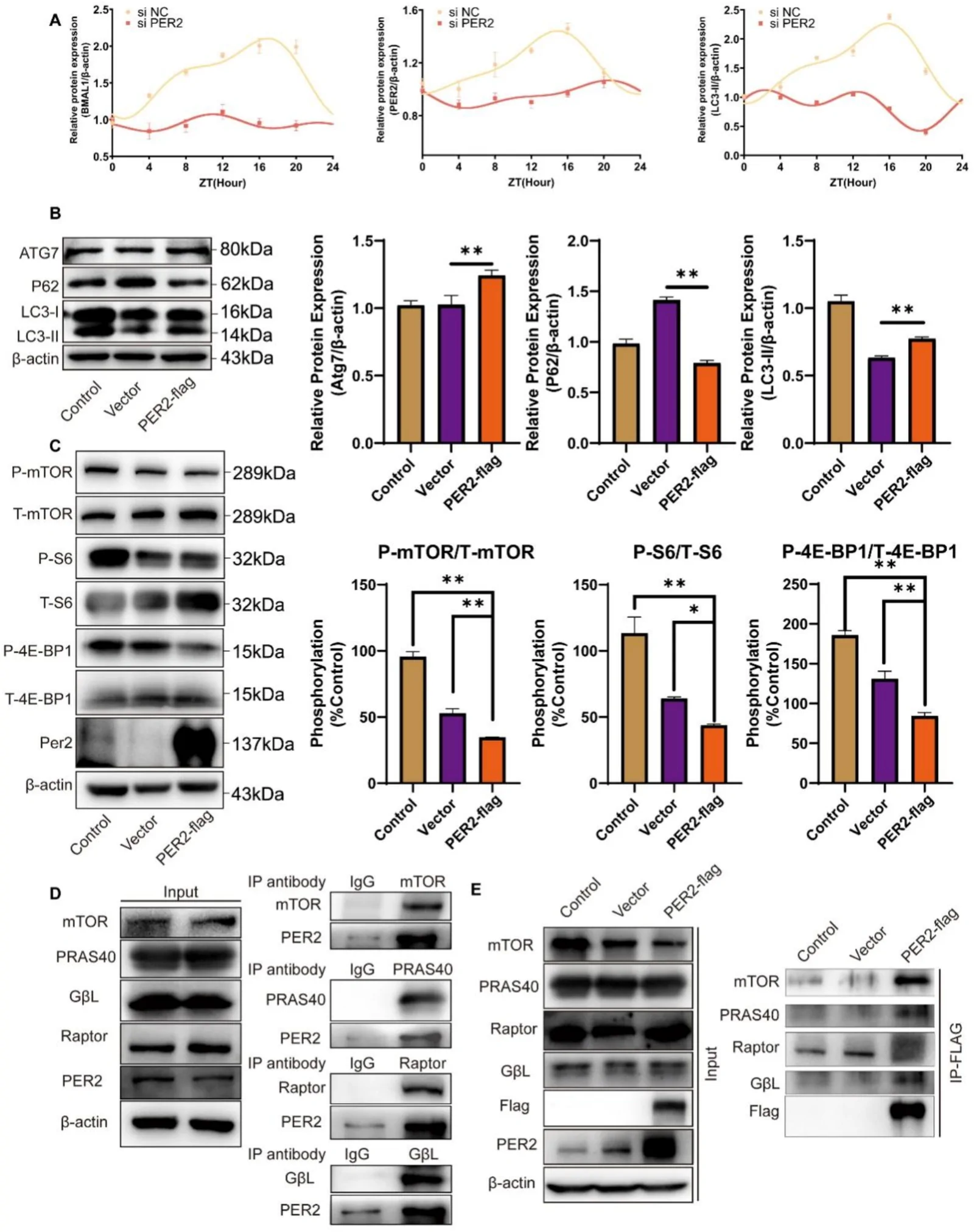
Interaction between PER2 and mTORC1. A. Rhythmic expression of BMAL1, PER2, and LC3-II protein in synchronized PER2-knocked down rat NP cells, n = 3. B. Expression of autophagy related protein after overexpression of PER2 in rat NP cells. Data are presented as mean ± SEM, n = 3. Differences were analyzed by two-way ANOVA. ** *P <* 0.01. C. phosphorylation of mTOR after overexpression of PER2 in NP cells. Data are presented as mean ± SEM, n = 3. Differences were analyzed by two-way ANOVA. ** P < 0.01. D. Co-Immunoprecipitation of mTOR, PRAS40, Raptor, and GβL in rat NP cells, n = 3. E. Co-Immunoprecipitation of PER2 in PER2-Flag-overexpressing rat NP cells, n = 3.

A Flag antibody was employed for co-immunoprecipitation (Co-IP) of recombinant rat PER2-Flag protein. Our results revealed that PER2-Flag interacts with each component of the mTORC1 complex in rat NP cells (Fig. 2D). Furthermore, using antibodies against mTOR, proline-rich Akt substrate of 40 kDa (PRAS40), Raptor, and GβL, we successfully co-immunoprecipitated the mTORC1-associated proteins and confirmed the binding of PER2 (Fig. 2E). These findings suggest that circadian rhythm (CR) may regulate autophagy in NP cells through the PER2-mTORC1 pathway.

### Physiological and pathological oxygen level and rhythm in NP tissues

We then set out to find a Zeitgeber for the circadian rhythm and autophagy rhythm in NP cells. Previously obtained transcriptome data from degenerated versus healthy NP tissue revealed [11] that the top 10 altered Kyoto Encyclopedia of Genes and Genomes (KEGG) pathways in NP tissue from degenerative patients included the Hypoxia inducible factor-1 (HIF-1) signaling pathway (Fig. 3A and B). Clustering genes associated with the HIF-1 signaling pathway in KEGG analysis revealed that the gene egl-9 family hypoxia inducible factor 1 (EGLN1) was significantly upregulated in degenerative patients (Fig. 3C). These findings suggest that the hypoxia signaling pathway plays a crucial role in the degeneration process. Considering the upregulation of genes involved in oxygen sensing and HIF-1α degradation in degenerative patients, we speculated that the oxygen concentration of NP is upregulated in degenerative patients, we speculated that the oxygen concentration of NP is upregulated in degenerative patients, aligning with evidence that degenerated discs undergo increased oxygenation due to vascular ingrowth, activating PHDs and destabilizing HIF-1α to exacerbate degeneration [34]. In human normal NP tissue, clear HIF-1α staining was observed in cells from each section, while prolyl hydroxylase 2 (PHD2) staining was only present in part of the section (Fig. 3D); in the degenerated NP tissue, HIF-1α levels were restricted to part of the section, while PHD2 levels increased and were found in most cells of each section (Fig. 3D).

**Fig. 3:**
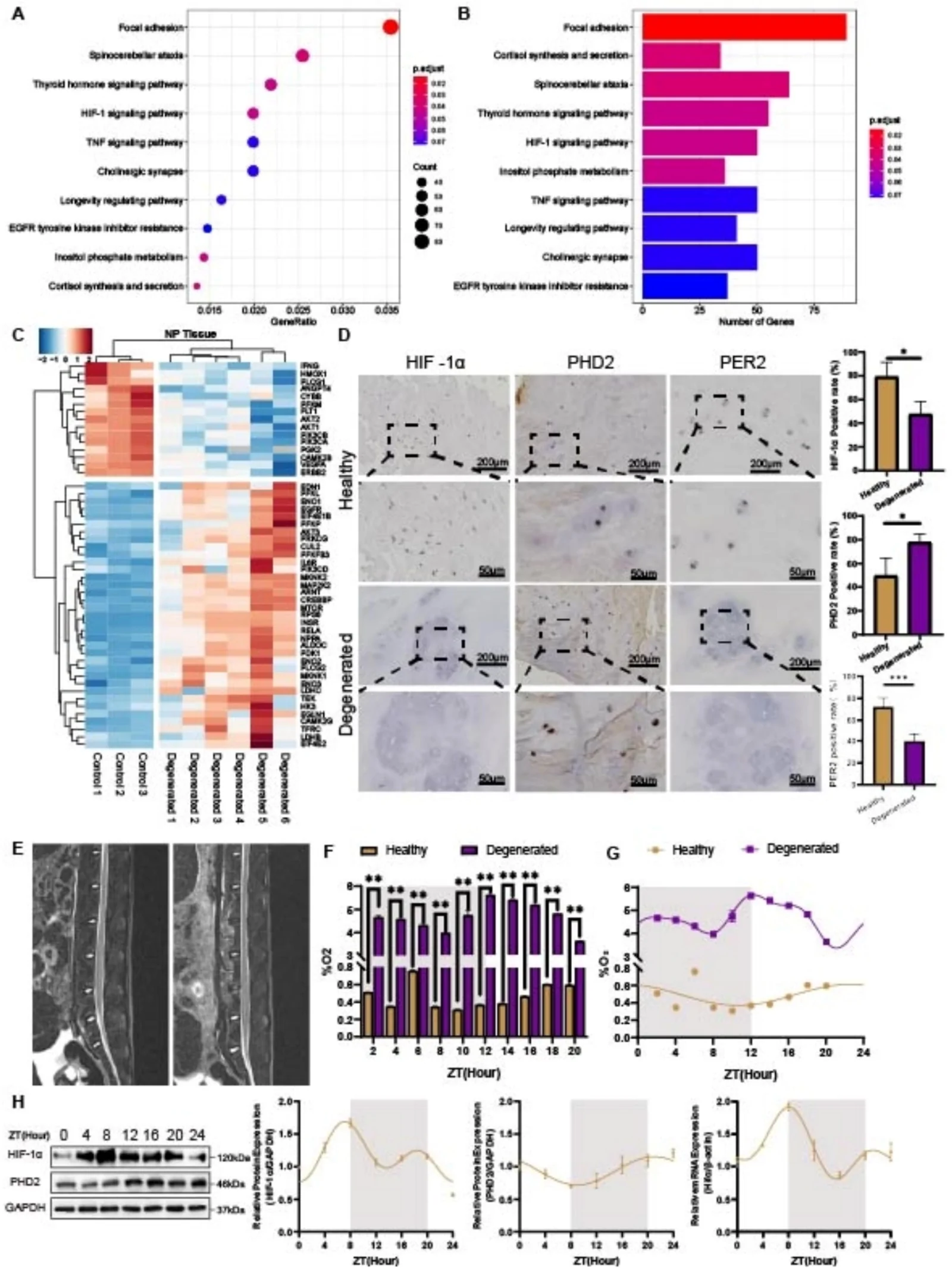
Oxygen changes in the nucleus pulposus tissues of healthy and degenerated intervertebral discs. A. Bubble map showing changes in KEGG pathways comparing the NP of human healthy and degenerated intervertebral discs. B. Bar chart showing changes in KEGG pathways comparing the NP of healthy and degenerated intervertebral discs. C. Heatmap of HIF-1α signaling pathway comparing the NP of healthy and degenerated intervertebral discs. D. Immunohistochemistry assay of HIF-1α, PHD2 and PER2 in the NP of human healthy and degenerated intervertebral discs, Data are presented as mean ± SEM, n = 6. Differences were analyzed by two-way ANOVA. *P < 0.05. E. MRI imaging of rabbit intervertebral discs before and after puncture modelling. F. Oxygen level of the NP in healthy and degenerated rabbit intervertebral discs. Data are presented as mean ± SEM, n = 3. Differences were analyzed by two-way ANOVA. ** *P <* 0.01. G. Oxygen level of the NP in healthy and degenerated rabbit intervertebral discs, n = 3. Data are superimposed with corresponding sine waves fitted through CircWave analyses. H. Rhythmic expression of HIF-1α and PHD2 protein and HIF-1α mRNA in the rat NP, n = 3. Data are superimposed with corresponding sine waves fitted through CircWave analyses.

A rabbit punctured IDD model was used to evaluate changes in oxygen levels in NP. One-month after the procedure, T2-weighted MR images of the lumbar spine revealed IVD degeneration (Fig. 3E). The oxygen concentration in healthy IVDs over a 24-hour period was significantly lower than that in degenerated IVDs. Specifically, the oxygen levels in healthy rabbit IVDs were maintained between 0.3 % and 0.8 %, whereas in degenerated rabbit IVDs, the oxygen concentration ranged from 3 % to 6 % (Fig. 3F). These results are consistent with in vivo measurements, which demonstrate significantly higher oxygen levels in degenerated discs compared to healthy ones in animal models [35]. Fluctuations in oxygen concentration between the healthy and degenerated groups followed a circadian rhythm (CR) (Fig. 3G), suggesting that hypoxia acts as a Zeitgeber in the NP microenvironment. To further investigate this, NP tissue from caudal IVDs of rats maintained under a 12:12 h light/dark (LD) cycle was extracted to assess the rhythmic expression of hypoxia-related genes and proteins. The mRNA levels of HIF-1α, as well as the protein levels of HIF-1α and PHD2, exhibited rhythmic oscillations (Fig. 3H). These findings are consistent with previous evidence showing that circadian clock genes, such as Bmal1, regulate HIF-1α transcriptional activity in NP cells, thereby influencing hypoxic responses and potentially alleviating the progression of intervertebral disc degeneration (IDD) [36]. Therefore, we suggested that NP cells respond to environmental changes in the oxygen content.

### Hypoxia reverses the disruption of the CR and autophagy rhythm induced by inflammation via the HIF-1α or PER2 pathway

Accumulating evidence suggests that local inflammation contributes to IDD progression, partly through the actions of proinflammatory cytokines such as IL-1β, which impair mitochondrial function and disrupt cellular homeostasis in NP cells [37]. We found that 10 ng/mL IL-1β can disrupt the CR of NP cells [8]. After 20 h of synchronization, NP cells were treatment with IL-1β for 24 h. The treatment interfered with both autophagy and circadian rhythms (Fig. 4), supporting previous observations that inflammation-related pathways, including NLRP3/IL-1β activation, disrupt circadian regulation in degenerated NP mesenchymal stem cells. Such disruptions, however, can be alleviated by circadian modulators like SR9009 through NR1D1-dependent signaling [38]. In this process, there was rhythmic mTOR phosphorylation after Dex synchronization, which was lost after IL-1β treatment (Fig. 4C).

**Fig. 4:**
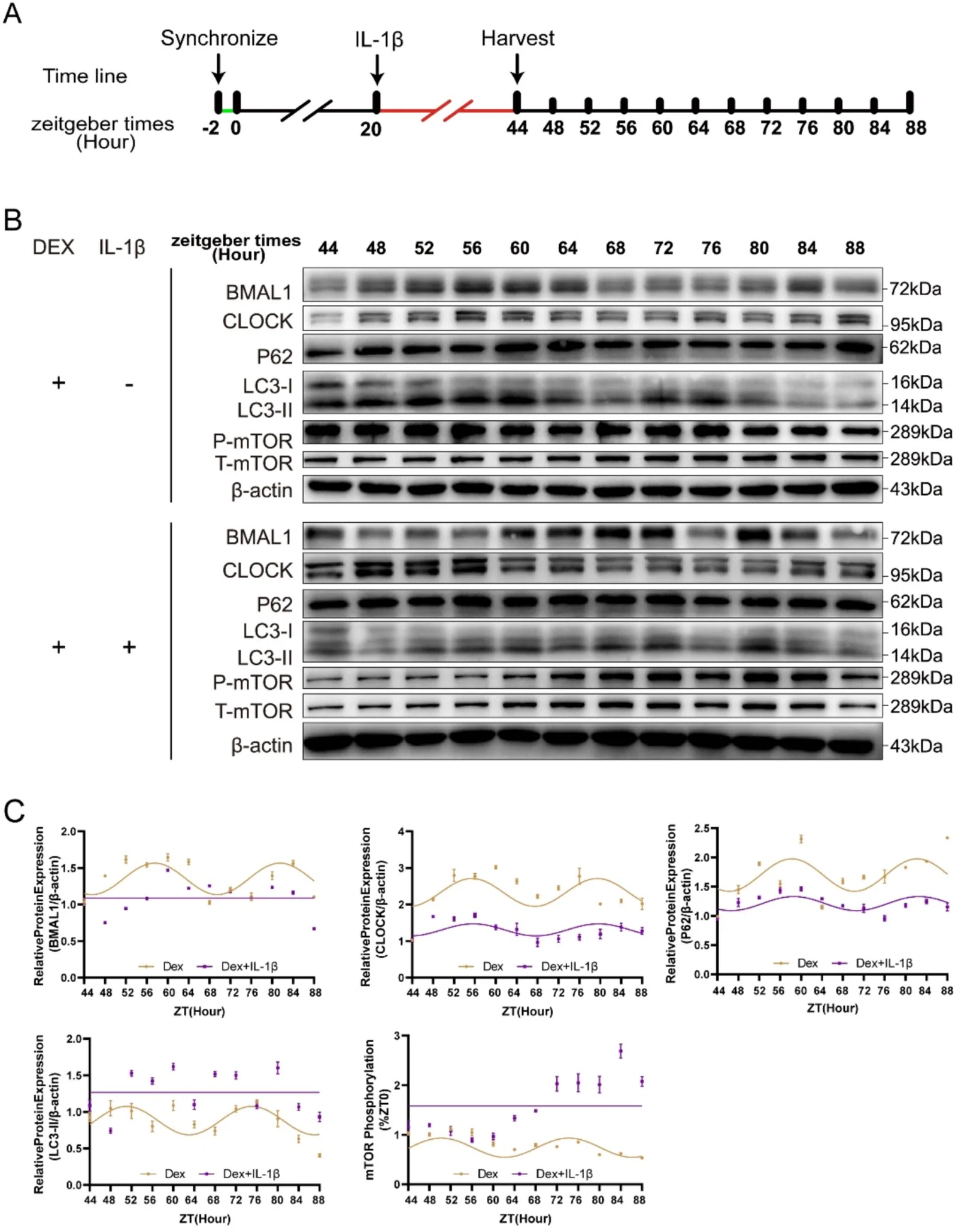
Disruption of circadian rhythm of clock and autophagy related proteins under inflammatory stimulator. A. Timeline of IL-1β treatment of synchronized rat NPs. B. Expression of BMAL1, CLOCK, P62, and LC3-II protein, and phosphorylation of mTOR in IL-1β-treated synchronized rat NP cells, n = 3. C. Data are superimposed with corresponding sine waves fitted through CircWave analyses.

Given that rat NP cells display a circadian rhythm (CR) under avascular conditions, we speculated that oxygen content may be a determinant of peripheral CR maintenance. This speculation aligns with findings that hypoxia preserves NP cell metabolic health via HIF-1α–mediated glycolysis and mitochondrial regulation, and that circadian regulators like BMAL1 influence HIF-1α activity to promote disc homeostasis [39]. Inflammatory factors were used to destroy the circadian clock of rat NP cells after synchronization. MetaCycle analysis revealed that BMAL1 and CLOCK exhibited robust circadian oscillations with a period of 24 h, while LC3-II maintained rhythmicity with a period of 25 h and amplitude of 0.24. The presence of IL-1β caused slight shifts in phase and amplitude of these proteins, with P62 showing little rhythmicity under the combined treatment. The NP cells were then placed in a hypoxic environment, or treated with CoCl_2_ to simulate a hypoxic environment, for 12 h to examine whether hypoxia can restore the CR. We found that the intermittent hypoxia restored the disrupted CR and autophagy rhythm induced by IL-1β (Fig. 5A, Fig. S5). MetaCycle analysis revealed that BMAL1 and CLOCK exhibited robust rhythmic oscillations under Dex, with peak times at ZT 16 and ZT 12, respectively, while LC3-II also maintained rhythmicity, with peaks at ZT 10 and an amplitude of 0.2004. The addition of IL-1β + 1 % O_2_ significantly dampened the rhythmicity, reducing BMAL1 and CLOCK's amplitude, and shifting LC3-II's peak to ZT 19. IL-1β abolished the Dex-synchronized circadian oscillation of HIF-1α in NP cells, markedly reducing rhythmic amplitude and phase coherence (Fig. S5C–E). Prolonged hypoxia (1–14 d) produced no overt cytotoxicity, with proliferation and live-cell imaging comparable to normoxia (Fig. S5F–G). Furthermore, to investigate the regulation of PER2 by hypoxia, we first compared its expression under physiological hypoxia (1 % O_2_) and chemical hypoxia induced by CoCl_2_. As shown in Fig. 5B, following HIF-1α silencing, PER2 expression was upregulated under 1 % oxygen concentration and CoCl_2_ treatment conditions compared to their respective si NC controls. These findings align with previous reports that hypoxia enhances PER2 expression via HIF-1α–driven transcriptional activation, in part through direct binding to promoter regions that reinforce circadian oscillations [33]. This design allowed us to isolate the effect of HIF-1α from oxygen level changes, demonstrating that the CoCl_2_-induced PER2 upregulation is indeed mediated by HIF-1α. To further examine the role of HIF-1α and PER2 in mediating hypoxia to restore CR, HIF-1α and PER2 were knocked down and cells were placed under a hypoxic environment to determine whether the CR could be restored. We found that the CR of NP cells with *Hif1α* or *PER2* knocked down could not be restored under a hypoxic microenvironment (Fig. 5C, Fig. S6), further suggesting that HIF-1α-mediated regulation of PER2 was involved in the re-synchronization. MetaCycle analysis (Fig. S6B) revealed that si PER2 and si HIF-1α induced phase shifts in key proteins, with BMAL1 maintaining a peak at ZT 18 and CLOCK showing a delayed peak at ZT 12 under si HIF-1α, while LC3-II and P62 exhibited altered rhythms in the knockdown conditions. The presence of si HIF-1α altered the amplitude and phase of the rhythmic proteins.

**Fig. 5:**
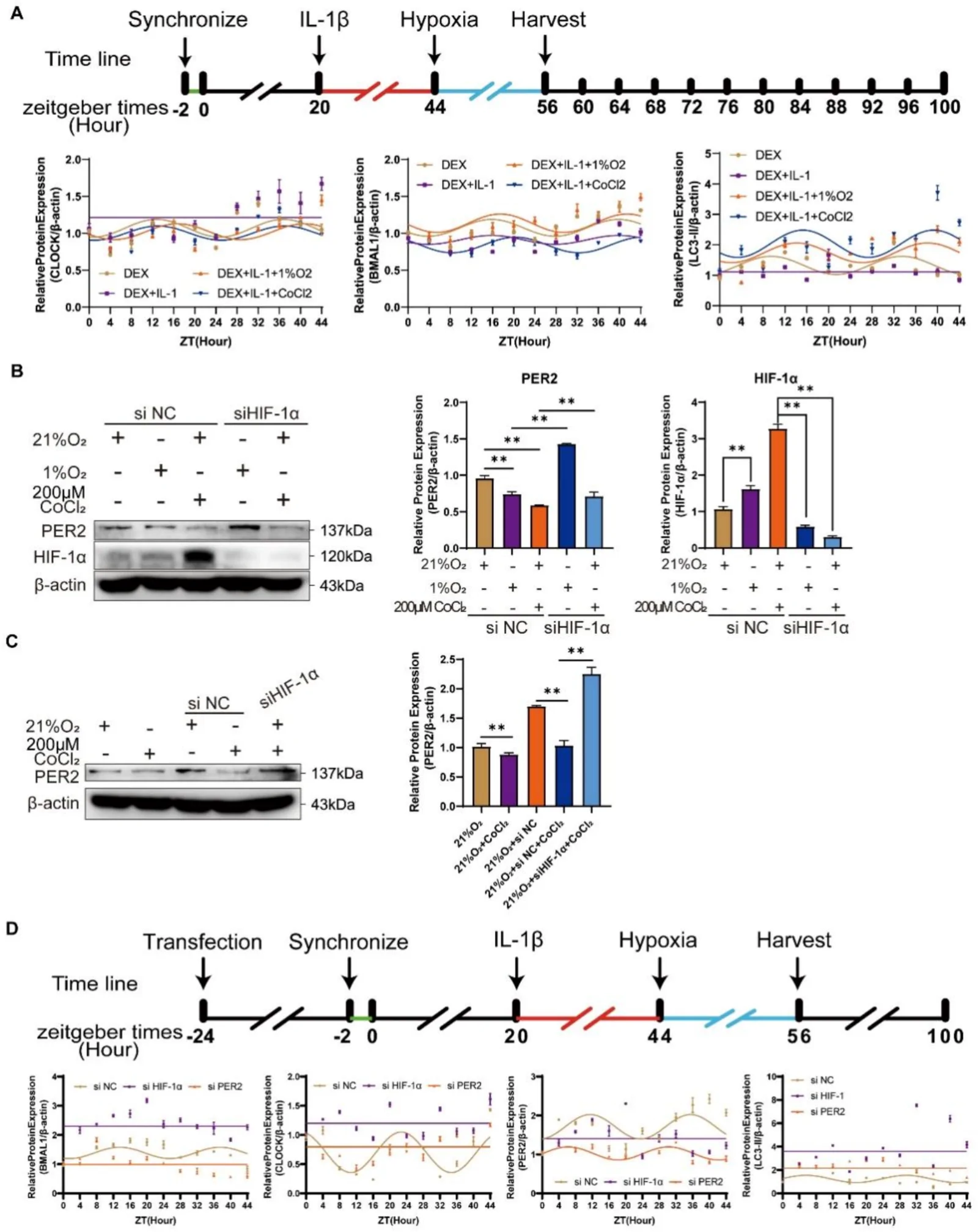
Hypoxia and cobalt chloride reverse the disruption of the circadian rhythm via HIF-1α dependent pathway. A. Rhythmic expression of BMAL1, CLOCK, and LC3-II protein in hypoxia-treated circadian rhythm-disrupted rat NP cells, n = 3. Data are superimposed with corresponding sine waves fitted through CircWave analyses. B. PER2 expression of hypoxia-treated rat NP cells. Data are presented as mean ± SEM, n = 3. Differences were analyzed by two-way ANOVA. ** *P <* 0.01. C. Expression of BMAL1, CLOCK, PER2, and LC3-II protein in hypoxia-treated HIF-1α/PER2 siRNA-transfected rat NP cells. Data are presented as mean ± SEM, n = 3. Differences were analyzed by two-way ANOVA. ** *P <* 0.01. D. Data are superimposed with corresponding sine waves fitted through CircWave analyses.

### Hypoxia restored the metabolism imbalance induced by autophagy inhibition and CR disruption

A rat model with a disrupted CR was developed and used to analyze the expression of matrix-and autophagy-related proteins in IVDs. We found that shifted rats exhibited more severe degeneration, characterized by increased cell vacuolization, reduced Aggrecan, and elevated cytoplasmic P62 in the shifted group (Fig. 6A). These changes suggest impaired autophagy in the affected IVDs and are consistent with reports that circadian rhythm disruption compromises mitochondrial homeostasis and autophagy, resulting in aggrecan loss and exacerbated degeneration in rat IDD models. Such effects can be alleviated by circadian regulators like melatonin through PGC-1α–dependent pathways [30]. In cells treated with KL001, collagen II levels decreased while matrix metalloproteinase (MMP)-3 levels increased (Fig. 6B). Thus, NP cells 24 h after Atg7 siRNA transfection, or bafilomycin A1 addition 24 h after synchronization, were used to mimic the damping of the autophagic CR. Reverse Transcription-Polymerase Chain Reaction (RT-PCR) and western blot analysis of the ZT48 point revealed that inhibition of autophagy attenuated anabolism and activated catabolism (Fig. 6C and D). This observation is consistent with previous findings that autophagy inhibition, whether through bafilomycin treatment or Atg7 ablation [40], reduces the expression of collagen II and aggrecan, while simultaneously upregulating MMP-13, which promotes intervertebral disc degeneration (IDD) features in NP cells. These results suggest that the regulation of autophagy plays a crucial role in maintaining the balance between anabolism and catabolism in NP cells.

**Fig. 6:**
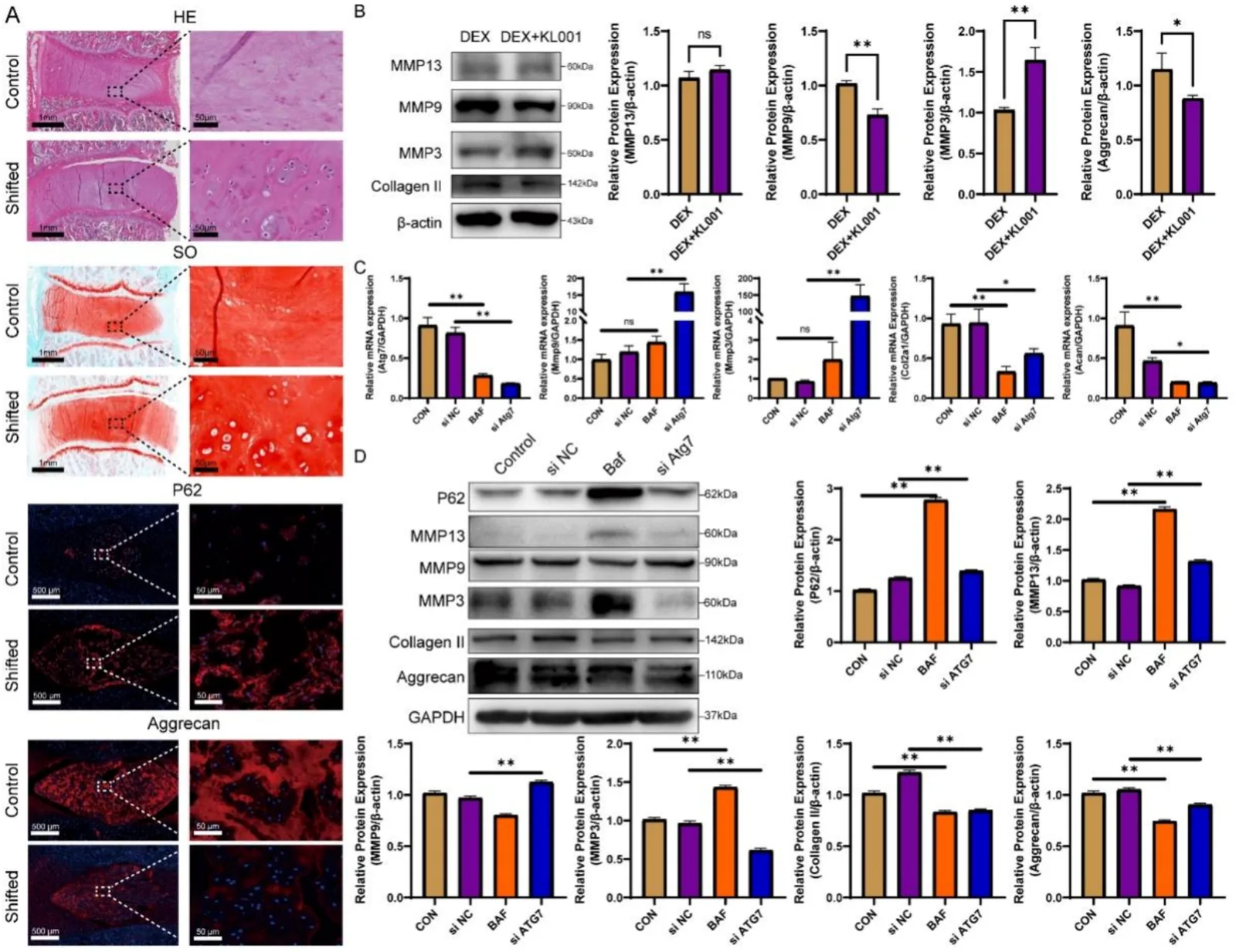
Circadian rhythm disruption and autophagy dysfunction resulted in catabolism of extracellular matrix. A. Hematoxylin-eosin staining, Safranin O-Fast Green Staining, and Immunofluorescence analysis of P62 and Aggrecan of the NP from control and shifted rat models, n = 3. B. Protein expression of MMP-13, −9, −3 and Collagen II compared with β-actin in KL001-treated synchronized NP. Data are presented as mean ± SEM, n = 3. Differences were analyzed by two-way ANOVA. ** *P <* 0.01. C. Expression of *ATG7*, *MMP9*, *MMP3*, *COL2A1*, and *ACAN* mRNA in autophagy inhibited synchronized NP. Data are presented as mean ± SEM, n = 3. Differences were analyzed by two-way ANOVA. * *P* < 0.05, ** *P* < 0.01. D. Relative expression of P62, MMP13, MMP9, MMP3, Collagen II, and Aggrecan protein compared with glyceraldehyde-3-phosphate dehydrogenase (GAPDH) in autophagy inhibited the synchronized NP. Data are presented as mean ± SEM, n = 3. Differences were analyzed by two-way ANOVA. ** *P <* 0.01.

Since HIF-1α expression in NP cells can be regulated by oxygen tension, we evaluated the effect of HIF-1α on circadian clock genes in NP cells. We analyzed anabolism and catabolism in ZT100 NP cells treated as shown in Fig. 5A. Hypoxia reversed the IL-1β-induced decrease in collagen II and the increase in MMP-3 and MMP-13 (Fig. 7A), suggesting that the hypoxic environment restored the autophagy rhythm and disrupted the balance between anabolism and catabolism in NP cells. This observation aligns with mechanisms where hypoxia-preconditioned exosomes induce mitophagy via BNIP3 [41], thereby alleviating intervertebral disc degeneration (IDD) and helping restore matrix integrity. Although IL-1β is a potent agent known to disrupt circadian rhythm in NP cells, it may also influence matrix anabolism and catabolism, rendering subsequent interventions unreliable. To address this, we investigated the effects of KL001 on matrix synthesis and degradation. We found that KL001 modulated matrix anabolism and catabolism in a time-dependent manner (Fig. S7A, B), with these effects being reversed upon KL001 withdrawal (Fig. S7C–E). IL‑1β had a dual effect on the extracellular matrix, simultaneously disrupting circadian rhythms and activating inflammatory pathways. This combined action markedly increased MMP13 and MMP3 expression while reducing type II collagen and aggrecan. By contrast, neither the cryptochrome‑specific inhibitor KL001 nor PER2 knockdown reproduced this pronounced degradation of the matrix, indicating that IL‑1β‑induced damage is not purely a circadian effect but also involves a substantial non‑rhythmic inflammatory component (Fig. S7F,G). KL001 was used as a substitute for IL-1β to intervene in NP cells prior to 12 h of hypoxic stress (Fig. 7B). At the ZT116 time point, we observed changes in matrix anabolism and catabolism, and found similar intervention effects: MMP-3 and MMP-9 levels were reduced, while Aggrecan levels increased under hypoxic conditions (Fig. 7C and D). Given that inhibiting autophagy in the presence of rhythmic disruption promotes matrix catabolism, 100 nM rapamycin was introduced to activate autophagy following rhythm disruption (Fig. 7E). We found that MMP-3 and MMP-9 levels decreased, while collagen II levels increased (Fig. 7F–G), suggesting that maintaining active autophagy is crucial for sustaining IVD matrix metabolism through the circadian rhythm. This builds on previous observations that rapamycin enhances autophagy flux to counteract degeneration, with potential links to circadian-regulated mTOR signaling, which plays a key role in metabolic homeostasis [42].

**Fig. 7:**
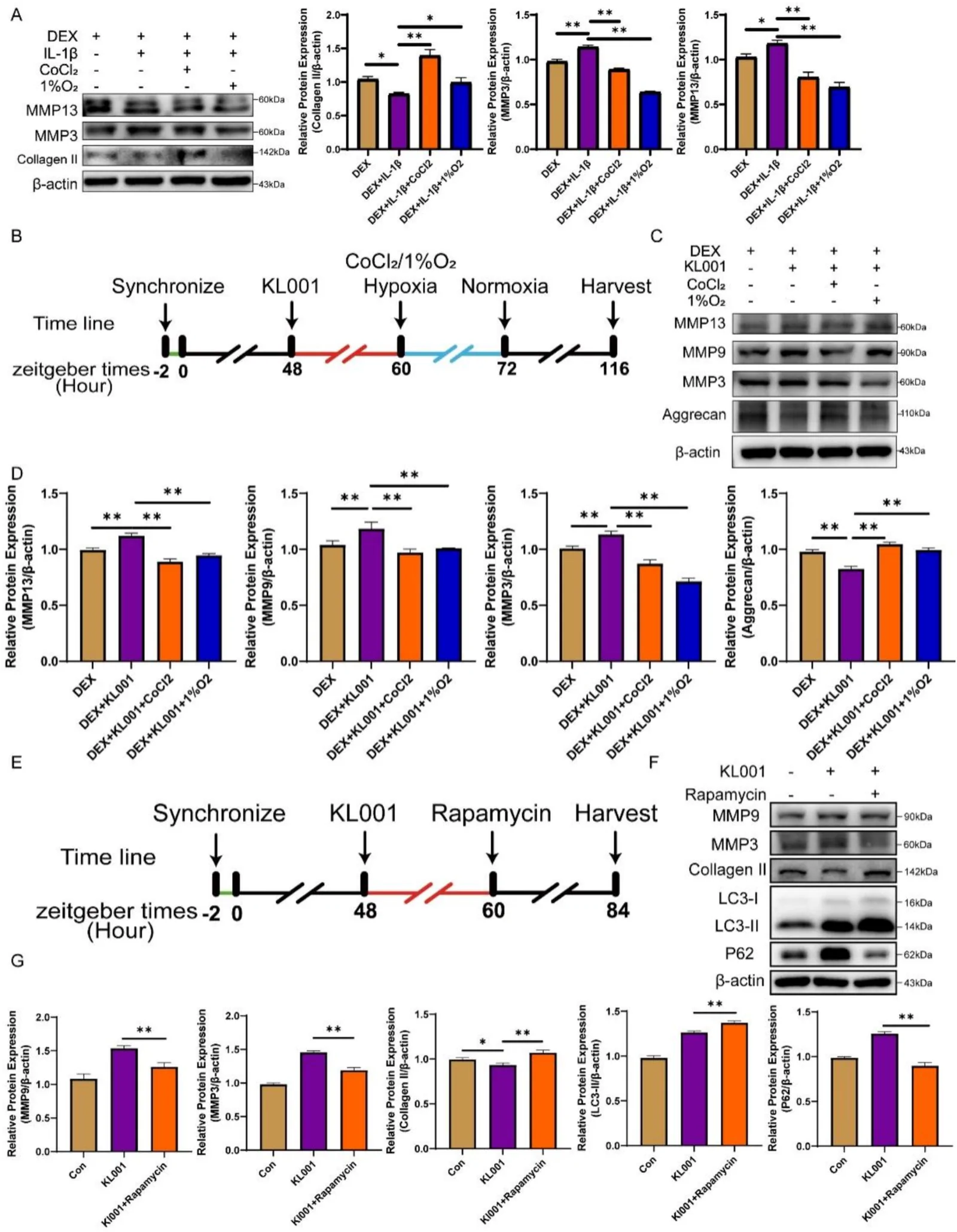
Hypoxia, CoCl_2_ and autophagic stimulator promoted the rebalance of extracellular matrix catabolism. A. Expression of MMP-13, −3, and Collagen II in hypoxia-treated KL001-treated synchronized NP. Data are presented as mean ± SEM, n = 3. Differences were analyzed by two-way ANOVA. ** *P <* 0.01. * *P* < 0.05, ** *P* < 0.01. B. Timeline of hypoxia and normoxia treatment following the inhibition of rhythm by KL001. C, D. Relative expression of MMP-3, −9, −13, and Aggrecan protein compared with β-actin in NP cells treated by time, as shown in B. Data are presented as mean ± SEM, n = 3. Differences were analyzed by two-way ANOVA. * *P <* 0.05, ** *P* < 0.01. E. Timeline of Rapamycin treatment following the inhibition of rhythm by KL001. F, G. Relative expression of MMP-3, −9, −13, and Collagen II protein compared with β-actin in NP cells treated over time, as shown in E. Data are presented as mean ± SEM, n = 3. Differences were analyzed by two-way ANOVA. ** *P* < 0.01.

### HIF-1α binds the PER2 promoter under hypoxia

Genome-wide profiles show a marked increase and sharpening of HIF-1α signal at peak centers under hypoxia (Fig. S9A). At the PER2 locus, genome-browser tracks display a discrete hypoxia-inducible peak across a ∼ 2.5 kb region that spans the proximal promoter, whereas signal is minimal in normoxia (Fig. S9B). This pattern is consistent with direct HIF-1α binding at the PER2 promoter. Genes associated with hypoxia-specific peaks are enriched for biological processes related to DNA damage responses, intrinsic apoptotic signaling, kinase regulation, and transcriptional responses to stress. Pathway analysis further highlights glycolysis and gluconeogenesis, HIF-1 signaling, AMPK and mTOR pathways, Notch signaling, and related metabolic and cancer pathways (Fig. S9C–D). Together, these data support a model in which hypoxia enables HIF-1α binding to the PER2 promoter, providing a mechanistic link between hypoxic signaling and PER2 transcription.

## Discussion

The disruption of peripheral circadian rhythm (CR) is a contributing factor to intervertebral disc degeneration (IDD) [8]; however, the mechanisms for maintaining CR stability in healthy NP cells remain poorly understood. Moreover, the peripheral CR influences downstream signaling pathways within the IVD, thereby regulating the degeneration process. The findings of this study demonstrate that the in vivo RNA expression of autophagy-related genes and proteins, along with rhythm genes, exhibits a 24-hour oscillatory pattern. Upon synchronization of human and rat NP cells with Dex in vitro, autophagy-related proteins in NP cells also displayed rhythmic expression. This rhythmicity was governed by the periodic phosphorylation of mTOR, which is regulated by PER2 in NP cells, thus contributing to the observed process.

Drawing inspiration from the alterations in the HIF-1 signaling pathway in NP tissue of patients with IDD, we observed that degenerated NP tissue exhibited a higher oxygen concentration compared to healthy NP tissue. Furthermore, the partial pressure of oxygen in normal NP tissue followed a 24-hour circadian rhythm (CR), with NP cells responding to fluctuations in oxygen levels. By modulating HIF-1α expression, our data suggest that HIF-1α negatively correlates with PER2 expression. These findings suggest that changes in oxygen concentration influence the CR of NP cells through the interaction between HIF-1α and PER2, thereby linking the hypoxic environment to circadian regulation in NP cells.

The integration of the circadian rhythm (CR) with behavior and the environment is crucial for maintaining normal physiological processes. As a key mechanism in metabolism, the circadian regulation of autophagy has been identified in tissues such as muscle, adipose, and liver. This process is modulated by the rhythm of eating and fasting and plays a protective role in both metabolic health and longevity, safeguarding cellular function and enhancing resilience against stressors [43]. In the hippocampus, the circadian rhythm (CR) of the autophagy-related protein LC3-II is closely linked to sleep patterns. Fragmented sleep disrupts the rhythmic expression of LC3-II, and even after sleep is restored, the expression of LC3-II does not return to its baseline oscillation levels. This suggests that sleep disturbances can have lasting effects on autophagic processes, potentially influencing cellular homeostasis and function [44]. In the outer retina, basal levels of autophagy are regulated by changes in circadian light input [[45], [46], [47]]. In addition, cyclic lighting activates autophagy in the neural retina [48]. Our study found that NP cells exhibit autophagy regulated by the CR.

The metabolic abnormalities observed in NP cells are likely a consequence of prolonged disruption of the circadian rhythm, which accumulates over time. This aligns with studies showing that circadian misalignment can lead to significant metabolic dysfunctions, such as impaired glucose/insulin tolerance and cardiometabolic issues. Mechanistically, upregulated miR-210 in human degenerated NP cells (NPCs) correlates with disc degeneration severity and targets autophagy-related gene 7 (ATG7) via 3′ UTR binding, inhibiting autophagic flux (reduced LC3-II, increased p62) and promoting extracellular matrix (ECM) degradation through elevated MMP-3/13 expression and reduced Col II/Aggrecan levels, as shown in miR-210 mimic/inhibitor and ATG7 knockdown experiments [49]. Additionally, BMAL1, a core circadian clock protein, regulates autophagy and mitophagy by modulating the PINK1/Parkin pathway. Disruption of BMAL1 leads to increased oxidative stress (ROS) and accelerated senescence in NP cells [50]. These findings suggest that circadian rhythm disruption results in long-term alterations in both autophagy and metabolism, contributing to the degeneration of NP cells.

The inflammatory environment plays an important role in destroying the CR [8] and is an important phenomenon in the process of IDD [51,52]. Therefore, the induction of an inflammatory environment to destroy the NP CR is closer to the physiological environment. However, the phosphorylation of mTOR is also affected by inflammatory factors [53], and the use of IL-1β may not fully reflect the influence of CR on autophagic activity. As a stabilizer of Cry protein, KL001 inhibits the CR by preventing the degradation of Cry protein [54]. After directly destroying the CR, the autophagy rhythm of the NP cells was also destroyed. This was consistent with the results obtained following the treatment of synchronous NP cells with IL-1β, confirming that the autophagy rhythm is coupled to the CR.

Mechanistically, IL-1β activates NF-κB/RELA, which binds the BMAL1 transactivation domain to repress BMAL1:CLOCK E-box transcription (and additionally downregulates the SIRT1–BMAL1 axis), a convergence that explains why IL-1β abolishes PER2/BMAL1 rhythms in IVD and cartilage models [55].

The high-oxygen environment in degenerative IVDs may arise from neovascularization, which disrupts the low-oxygen homeostasis of NP cells, leading to oxidative stress and matrix alterations. Additionally, endplate calcification or fissures may enhance oxygen diffusion [56]. We propose that high oxygen tension could act as an initiating factor for the HIF-1α-mediated pathway by destabilizing HIF-1α through prolyl hydroxylase-mediated degradation, shifting NP cells from glycolytic to oxidative metabolism, thereby promoting cell death and degeneration [57]. This destabilization of HIF-1α may interact with circadian pathways, as molecular clock components like BMAL1 and RORα regulate HIF-1α function in hypoxic niches. Hyperoxic conditions are known to alter both HIF-1α and circadian gene expression, potentially exacerbating degenerative processes [58]. Therefore, the high-oxygen environment may act synergistically with inflammation as an initiator of IDD, warranting further investigation into this interplay.

The difference in oxygen concentration between healthy and degenerated NP cells has not previously been detected in patients [12]. This may be explained by changes in the evaluation of IVD degeneration [12]. The level of degeneration has previously been evaluated by gross photos of surgical specimens, a process lacking in scientific detection [59]. By confirming the level of IVD degeneration by MR, we were able to reveal, for the first time, the oxygen content in the degenerated IVD and its biological consequences [60].

There exists a significant correlation between oxygen concentration and metabolism in NP cells. Oxygen consumption, glucose levels, and lactic acid concentrations in deep NP cells were notably different from those in outer regions. Glycolysis, typically occurring under anaerobic conditions, is prominent in these deeper NP cells, where oxygen concentration decreases significantly from the outer fibrous annulus to the inner depths of the NP. Given the avascular nature of the intervertebral disc (IVD), which limits the entry of nutrients and signaling molecules from the serum, oxygen—being a small, low-molecular-weight gas molecule—plays a crucial role in regulating cellular metabolism. Hypoxia, therefore, may be vital for the exchange of information between the IVD and the rest of the body, influencing both metabolic and cellular processes in this unique environment [61,62]. The results of this study demonstrated that the oxygen content within the NP exhibits rhythmic fluctuations, with these fluctuations being more pronounced in degenerated NP tissue. This pattern corresponds to the pathological changes observed during NP degeneration as well as the physiological rhythm of blood oxygen levels. Alterations in oxygen concentration effectively regulate the HIF-1 signaling pathway in NP cells, leading to the restoration of circadian rhythm (CR) in these cells. These findings underscore the physiological significance of circadian variations in oxygen concentration within the NP, highlighting their role in maintaining cellular function and metabolism in the intervertebral disc.

Previous studies have shown that the biological rhythm of NP cells can be affected by changes in the body temperature of animals [8,63]. A previous study demonstrated that temperature could regulate the CR of NP cells through time sequential transcriptome sequencing of rat and mouse NP cells and culturing under variable temperature; however, the pathway through which temperature affects the biological clock has not been elucidated [8,63]. In this study, we combined the biological clock and hypoxia-related pathways through the temporal detection of hypoxia genes in vivo and half-day hypoxia intervention in vitro, and examined the regulatory effect of the microenvironment on the biological clock.

HIF-1α presents temporal and spatial specificity in the regulation of PER2 [19]. In nuclear extracts from the liver, hypoxia induced PER2 expression at night, while hypoxia inhibited PER2 expression at night in nuclear extracts from the kidney and lung. In this study, hypoxia was found to regulate the biological clock by inhibiting the expression of PER2 in NP cells through HIF-1α. Although CUT&Tag sequencing technology identified hypoxia-dependent HIF-1α peaks in the upstream promoter region of the PER2 gene, confirming that hypoxia influences circadian rhythms through direct regulatory mechanisms, the interaction between HIF-1α and PER2 protein in NP cells warrants further investigation [64,65].

This study revealed that autophagic activity in NP cells follows a biological rhythm, which is regulated by the cells' intrinsic biological clock through the interaction between PER2 and mTOR. However, direct evidence remains lacking on whether restoring autophagy rhythms can maintain ECM homeostasis, underscoring the need for long-term, real-time monitoring experiments of autophagy rhythms and ECM metabolism. Additionally, the oxygen levels in degenerative IVDs are significantly higher than in healthy IVDs, with both healthy and degenerative discs exhibiting a circadian rhythm (CR) in oxygen content. Healthy IVDs respond effectively to diurnal fluctuations in the oxygen levels of the NP. Degeneration, however, induces the upregulation of hypoxia-related signaling pathways in the IVD, characterized by an increase in EGLN1/PHD2 and a decrease in HIF-1α. The hypoxic microenvironment plays a crucial role in synchronizing the CR of NP cells via the HIF-1α/PER2 pathway, highlighting the interplay between oxygen levels and circadian regulation in disc health and degeneration. The HIF-1α–PER2–mTOR/autophagy axis nominates concrete therapeutic directions in IDD: restoring core clock function (e.g., BMAL1/SIRT1/PGC-1α-linked) [66], leveraging clock-targeting miRNAs to enhance autophagic flux (hsa-let-7f-1-3p/BMAL1) [67], and implementing circadian-timed autophagy activation (mTOR/AMPK-directed) with disc-targeted delivery and time-of-day dosing.

## Ethics statement

This study was conducted exclusively on the invertebrate planarian Dugesia japonica. No vertebrate organisms were involved in this study.

## Declaration of competing interest

The authors declare that they have no known competing financial interests or personal relationships that could have appeared to influence the work reported in this paper.
