# Supplementary material for: Hypoxia inhibits intervertebral disc degeneration by maintaining autophagy circadian rhythm via the HIF1α-PER2-mTOR pathway
Source: J Adv Res. 2025 Dec 28;88:759–74. doi: 10.1016/j.jare.2025.12.036 (PMC13669551; doi:10.1016/j.jare.2025.12.036)
Supplement: Supplementary file 1 [file mmc1.docx]

**Supplementary information**

Supplementary methods

1.Identification of NP cells

NP cells were seeded on cell slides. When the NP cells had grown to cover 60–70% of the bottom area of the dish, the culture medium was discarded, the cells were washed three times with PBS, fixed with 4% paraformaldehyde for 20 min, and then washed three times for the following experiments.

1.1. Alcian blue staining

Alcian Blue 8GX (0.1 g) was added to a mixture of 80 mL absolute ethanol and 20 mL glacial acetic acid to prepare an Alcian blue dyeing working solution. The working solution was added dropwise to the slide, incubated at room temperature for 30 min, rinsed with tap water for 2 min until no staining solution remained, dried, and then neutral gum was used to mount the slide to visualize the staining results.

1.2. Toluidine blue staining

Toluidine blue dye solution (0.1%) was added dropwise on the slide, which was then incubated at room temperature for 10 min, washed with distilled water three times, 5 min each time, dried, and mounted with neutral gum to visualize the staining results.

1.3. Immunofluorescence staining of type 2 collagen

Cells were treated with 0.5% Triton-100 diluted in PBS for 30 min to punch the cells, washed cells three times with PBS, blocked the cells with PBST-diluted protein-free fast blocking solution for 10 min, and then incubated with a Collage II primary antibody (1:200 dilution) overnight at 4 °C. The cells were washed with PBST three times for 5 min each, Cy3-labeled goat anti-rabbit secondary antibody diluted in PBST was added, incubated for 1 h in the dark, washed three times with PBST, 5 min each time, using 0.1 μg/mL. After staining with DAPI staining solution for 20 min, glycerin was used to mount the slide, which were then visualized under a fluorescence microscope.

1.4. Oxygen concentration

In this study, oxygen levels in the intervertebral disc (IVD) were measured using a Clark-type microelectrode and fiber-optic oxygen probe (OxyLite). The probes were calibrated before each experiment using a two-point calibration method: 0% O₂ (sodium sulfite solution) and 100% O₂ (air-saturated water). Calibration was verified post-experiment to ensure accuracy, with data showing <5% drift in 24-hour recordings. To minimize tissue damage, probes were inserted using a protective cannula to reduce trauma, with a lateral aperture used for oxygen measurements. The probes were allowed to stabilize for at least 60 seconds before any readings were taken to avoid insertion artifacts. The animals were anesthetized using isoflurane (5% for induction, 2% for maintenance), and mechanical ventilation was maintained throughout the procedure with fixed FiO₂ (21%) to ensure stable oxygen levels. Respiratory gases (end-tidal CO₂, FiO₂) and arterial blood gases were monitored to confirm proper ventilation. Oxygen levels in the IVD were measured continuously, and the influence of anesthesia and ventilation was accounted for by ensuring consistent parameters throughout the measurements. Drift in oxygen readings was monitored continuously, and any readings with significant post-measurement drift (>5%) were excluded from analysis.

1.5. Hypoxia

Cells were exposed to hypoxic conditions either by incubation in a tri-gas incubator (Thermo Fisher Scientific, USA) set to 1% O₂, 5% CO₂, and 94% N₂ at 37 °C for 12 h, or by chemical hypoxia using cobalt chloride (CoCl₂; Sigma-Aldrich, USA). For gas-induced hypoxia, culture medium and plates were pre-equilibrated in the hypoxia chamber for at least 2 h, and once the oxygen level stabilized at 1% (±0.2%), cells were maintained continuously for 12 h before sample collection. For chemical hypoxia, a 100 mM CoCl₂ stock solution prepared in sterile water was diluted in complete medium to a final concentration of 200 μM, and cells were incubated under normoxic conditions (21% O₂, 5% CO₂) for 12 h. Control groups were maintained in normoxia or treated with vehicle (sterile water) under identical conditions. CoCl₂ was selected as a hypoxia mimetic because it inhibits prolyl-hydroxylase (PHD) activity and stabilizes HIF-1α, enabling rapid and reproducible induction of hypoxia signaling with straightforward handling and low cost.

1.6. Ethical Approval and Animal Experiments

The experimental design was approved by the Animal Protection Committee of Zhongshan Hospital Affiliated to Fudan University (IACUC#2023-168). The handling and use of animals followed the "Guidelines for the Care and Use of Laboratory Animals" of the National Department of Health. For this experiment, 50 4-week-old specific pathogen-free (SPF) male Sprague Dawley (SD) rats and 12 2.5 kg male New Zealand rabbits were purchased from Shanghai Jiesjie Experimental Animal Center and raised in the Experimental Research Center of Zhongshan Hospital Affiliated to Fudan University. Animals were anesthetized using isoflurane (2-3% in 100% oxygen) via inhalation until loss of consciousness. Euthanasia was performed using CO₂ inhalation, followed by cervical dislocation to confirm death. Pain management was provided using meloxicam (0.2 mg/kg, subcutaneously) once daily for 3 days post-surgery in rabbits.

1.7. CUT&Tag

CUT&Tag was performed under native conditions on nucleus pulposus cells in two experimental groups—normoxia and hypoxia—to profile HIF-1α chromatin occupancy. Cells were bound to concanavalin A beads, gently permeabilized, incubated with anti–HIF-1α, tethered to pre-loaded pA-Tn5, and subjected to on-bead tagmentation; released DNA was purified, PCR-indexed, pooled, and sequenced on an Illumina platform. Reads were adapter-trimmed and aligned to the reference genome, mitochondrial/blacklist regions and duplicates were removed, and peaks were called against IgG using SEACR (with MACS2 as a sensitivity check); biological replicates were evaluated for concordance, normalized coverage tracks were generated for visualization, and differential binding between hypoxia and normoxia was assessed with motif (HRE) and functional enrichment on hypoxia-induced sites. Reagents and antibodies are listed in the Key Resources Table.

Supplementary Table 1. Patient characteristics and Pfirrmann classification for histological assessment and cell culture.

| No. | Age | Gender | Diagnosis | Disc Level |  | Pfirrmann Grade |
| --- | --- | --- | --- | --- | --- | --- |
| 1 | 35 | Male | IVD herniation | L5–S1 |  | V |
| 2 | 48 | Female | Spine fracture | C5–6/C6–7 |  | II |
| 3 | 67 | Female | IVD herniation | L2–L3 |  | V |
| 4 | 77 | Female | IVD herniation | L4–L5 |  | IV |
| 5 | 33 | Male | Spine fracture | T11–T12/T12–L1 |  | II |
| 6 | 48 | Female | IVD herniation | L4–L5 |  | IV |
| 7 | 56 | Female | IVD herniation | L5–S1 |  | IV |
| 8 | 54 | Male | IVD herniation | L4–L5 |  | III |
| 9 | 52 | Female | IVD herniation | L4–L5 |  | IV |
| 10 | 22 | Female | IVD herniation | L4–L5 |  | II |

Supplementary Table 2. Animal disc height and Pfirrmann Grade.

| No. | Organism | Group | Disc Level | DHI | Pfirrmann Grade |
| --- | --- | --- | --- | --- | --- |
| 1 | Rabbit | Control | L4-L5 | 22 | I |
| 2 | Rabbit | Control | L4-L5 | 21 | I |
| 3 | Rabbit | Control | L4-L5 | 22 | I |
| 4 | Rabbit | Shifted | L4-L5 | 16 | III |
| 5 | Rabbit | Shifted | L4-L5 | 14 | IV |
| 6 | Rabbit | Shifted | L4-L5 | 17 | III |

Supplementary table 3 siRNA used in the experiments

| Target | Sequence |
| --- | --- |
| *Atg7* | 5'-GCAUCAUCUUUGAAGUGAA-3' |
| *PER2* | 5’-GCTATGAAGCTCCTAGAAT-3’ |
| *Hif1a* | 5’-TCGACAAGCTTAAGAAAGA-3’ |

Supplementary table 4 Primers used for RT-PCR

| Organism | Gene | Primers | |
| --- | --- | --- | --- |
| Human | *CLOCK* | Forward | GGCAAAATGTCATGAGCACTTA |
|  |  | Reverse | AGCCCTAACTTCTGCATAACTT |
| Human | *PER2* | Forward | CTTATTCACTGCCCGTGTTTC |
|  |  | Reverse | GGAAGGAATAACTGGGTAGCAT |
| Human | *Arntl* | Forward | TGCCACCAATCCATACACAGAAGC |
|  |  | Reverse | ATCTTCCCTCGGTCACATCCTACG |
| Human | *ATG5* | Forward | GATGGGATTGCAAAATGACAGA |
|  |  | Reverse | GAAAGGTCTTTCAGTCGTTGTC |
| Human | *ATG7* | Forward | TGTATAACACCAACACACTCGA |
|  |  | Reverse | GGCAGGATAGCAAAACCAATAG |
| Human | *MAP1LC3A* | Forward | CCTGGACAAGACCAAGTTTTTG |
|  |  | Reverse | GTAGACCATATAGAGGAAGCCG |
| Human | *SQSTM1* | Forward | TGATTGAGTCCCTCTCCCAGATGC |
|  |  | Reverse | CCGCTCCGATGTCATAGTTCTTGG |
| Human | *ACTB* | Forward | GGCCAACCGCGAGAAGATGAC |
|  |  | Reverse | GGATAGCACAGCCTGGATAGCAAC |
| Rat | *Clock* | Forward | GCGAGAACTTGGCGTTGAGGAG |
|  |  | Reverse | TGGAGGAGGCAGAAGGAGTTGG |
| Rat | *PER2* | Forward | GCTCGCCATCCACAAGAAGATCC |
|  |  | Reverse | CCTGCTCCACGGGTTGATGAAG |
| Rat | *Bmal1* | Forward | AGGACTTCGCCTCCACCTGTTC |
|  |  | Reverse | GCCCTCATTGTCTGGTTCACTGTC |
| Rat | *Atg5* | Forward | CTCAGCTCTGCCTTGGAACATCAC |
|  |  | Reverse | AAGTGAGCCTCAACTGCATCCTTG |
| Rat | *Atg7* | Forward | GTGAACCTCAGCGGATGTATGGAC |
|  |  | Reverse | CCAGCAGCAGGCACTTGACAG |
| Rat | *Map1lc3a* | Forward | TCGCCGACCGCTGTAAGGAG |
|  |  | Reverse | GGTTCACCAGCAGGAAGAAGGC |
| Rat | *Sqstm1* | Forward | AGTTCCAGCACAGGCACAGA |
|  |  | Reverse | ACAAATGCGTCCAGTCGTCA |
| Rat | *Actb* | Forward | CTAAGGCCAACCGTGAAAAGAT |
|  |  | Reverse | GACCAGAGGCATACAGGGACAA |
| Rat | *Hif1a* | Forward | TTCTCCAAGCCCTCCGAGTGTG |
|  |  | Reverse | GCGGTGGCAGTGACAGTGATG |
| Rat | *Egln1* | Forward | AGGCAACGGGACAGGCTACG |
|  |  | Reverse | CGAAGAATACCTCCGCTCACCTTG |
| Rat | *Col2a1* | Forward | ACGCTCAAGTCGCTGAACAACC |
|  |  | Reverse | ATCCAGTAGTCTCCGCTCTTCCAC |
| Rat | *Acan* | Forward | CTGATCCACTGTCCAAGCACCATG |
|  |  | Reverse | ATCCACGCCAGGCTCCACTC |
| Rat | *Mmp9* | Forward | CTACACGGAGCATGGCAACGG |
|  |  | Reverse | TGGTGCAGGCAGAGTAGGAGTG |
| Rat | *Mmp3* | Forward | CAGTCCTGCTGTGGCTGTGTAC |
|  |  | Reverse | AACCTCCATGCCAGCATCTTCTTC |

Supplementary table 5 Antibodies and dilutions

| Antibody | Source | IF/IHC | WB |
| --- | --- | --- | --- |
| Primary antibody | | | |
| Bmal1 (rabbit monoclonal) | Cell Signaling Technology #14020 |  | 1: 1000 |
| PER2 (rabbit polyclonal) | ABclonal #A3217 |  | 1: 1000 |
| CLOCK (rabbit monoclonal) | Abcam #ab3517 |  | 1: 2000 |
| LC3A/B (rabbit monoclonal) | Cell Signaling Technology #12741 |  | 1:500 |
| SQSTM1/p62 (rabbit polyclonal) | Serviceio #GB11239-1 | 1:200 | 1:500 |
| ATG7 (mouse monoclonal) | Proteintech #67341-1-Ig |  | 1:1000 |
| ATG5 (mouse monoclonal) | Proteintech #66744-1-Ig |  | 1:1000 |
| PHD2 (rabbit polyclonal) | Proteintech #19886-1-AP | 1:200 | 1:1000 |
| HIF-1α (rabbit monoclonal) | Cell Signaling Technology #14179 |  | 1:1000 |
| HIF-1α (rabbit polyclonal) | Bioss #bs-3927R | 1:200 |  |
| Raptor (rabbit polyclonal) | Proteintech #20984-1-AP |  | 1:1000 |
| mTOR (mouse monoclonal) | Proteintech #66888-1-Ig |  | 1:1000 |
| Phospho-mTOR (rabbit) | Cell Signaling Technology #5536 |  | 1:1000 |
| PRAS40 (rabbit polyclonal) | Cell Signaling Technology #2691 |  | 1:1000 |
| GβL(rabbit monoclonal) | Cell Signaling Technology #3274 |  | 1:1000 |
| S6 | Cell Signaling Technology #2217 |  | 1:2000 |
| Phospho-S6 | Cell Signaling Technology #4858 |  | 1:2000 |
| 4E-BP1 (rabbit monoclonal) | Cell Signaling Technology #9644 |  | 1:1000 |
| Phospho-4E-BP1 (rabbit monoclonal) | Cell Signaling Technology #2855 |  | 1:1000 |
| Collagen II (rabbit monoclonal) | Abcam #ab34712 | 1: 200 | 1: 200 |
| Aggrecan (rabbit polyclonal) | Abcam # ab36861 | 1: 200 | 1: 200 |
| MMP-3 (rabbit polyclonal) | Absin #abs135854 |  | 1:1000 |
| MMP-9 (rabbit polyclonal) | Absin #abs 133821 |  | 1: 1000 |
| MMP-13 (rabbit polyclonal) | Absin #abs136014 |  | 1: 1000 |
| β-actin (mouse monoclonal) | Abcam #ab8226 |  | 1: 1000 |
| GAPDH (rabbit monoclonal) | Abcam #ab181602 |  | 1: 2000 |
| Secondary antibody | | | |
| Cy3-labeled Goat Anti-Rabbit IgG(H+L) | BeyotimeA0516 | 1: 200 |  |
| Horseradish peroxidase - conjugated Peroxidase AffiniPure Goat Anti-Mouse IgG (H+L) | Jackson ImmunoResearch #115-035-003 | 1:500 | 1:5000 |
| Horseradish peroxidase - conjugated Peroxidase AffiniPure Goat Anti-Rabbit IgG (H+L) | Jackson ImmunoResearch #111-035-003 | 1:500 | 1:5000 |

**Fig. S1. Identification of nucleus pulposus cells.** **A.** Rat nucleus pulposus cells under a light microscope. **B.** Rat nucleus pulposus cells stained with toluidine blue. **C.** Rat nucleus pulposus cells stained with Alcian blue. **D.** DAPI staining of rat nucleus pulposus cells. **E.** Rat nucleus pulposus cells fluorescently stained with type II collagen **F.** Merge of D & E. **G.** Human nucleus pulposus cells under a light microscope. **H.** Human nucleus pulposus cells stained with toluidine blue. **I.** Human nucleus pulposus cells stained with Alcian blue. **J.** DAPI staining of human nucleus pulposus cells. **K.** Human nucleus pulposus cells fluorescently stained with type II collagen **L.** Merge of D & E.

**Fig. S2. Rhythmic expression of autophagy related proteins in nucleus pulposus cells.** **A.** Rhythmic expression of BMAL1, CLOCK, P62, and LC3-II in control and dexamethasone-synchronized rat nucleus pulposus cells, n=3. **B.** Rhythmic expression of BMAL1 and LC3-II in control and dexamethasone-synchronized human nucleus pulposus cells, n=3. **C.** Rhythmic expression of BMAL1, LC3-I, and LC3-II in control and dexamethasone-synchronized rat nucleus pulposus cells, n=3. **D.** MetaCycle analysis of rhythmic gene expression from panels A and C. **E.** MetaCycle analysis of data from Fig. 1D. **F.** Expression of PER2 protein after overexpression of PER2 in rat NP cells. Data are presented as mean ± SEM, n=3. Differences were analyzed by two-way ANOVA. ** P<0.01.

**Fig. S3. Autophagy rhythm and circadian rhythm disruption after KL001 treatment. A.** mRNA expression of Arntl and PER2 in KL001-treated rat nucleus pulposus cells. Data are presented as mean ± SEM, n=3. Differences were analyzed by two-way ANOVA. * P<0.05, ** P<0.01. **B.** Timeline of KL001 treatment in synchronized rat nucleus pulposus cells. **C.** Expression of BMAL1, CLOCK, ATG5, P62, and LC3-II in KL001-treated synchronized rat nucleus pulposus cells, n=3. **D.** MetaCycle-filtered circadian rhythmic gene expression in KL001-treated synchronized rat nucleus pulposus cells. **E.** Rhythmic protein expression of BMAL1, CLOCK, ATG5, and P62 in control and KL001-treated rat nucleus pulposus cells, shown by MetaCycle analysis.

**Fig. S4. Disruption of the circadian rhythm of autophagy after siPER2. A.** Verification of PER2 siRNA. Data are presented as mean ± SEM, n=3. Differences were analyzed by two-way ANOVA. ** P<0.01. **B.** Knock-down of PER2 inhibited autophagy. Data are presented as mean ± SEM, n=3. Differences were analyzed by two-way ANOVA. ** P<0.01. **C.** Knock-down of PER2 activated the mTOR signaling pathway. Data are presented as mean ± SEM, n=3. Differences were analyzed by two-way ANOVA. ** P<0.01. **D.** Timeline after PER2 siRNA transfection. **E.** Expression of BMAL1, CLOCK, PER2, and LC3-II protein in synchronized PER2 siRNA-transfected nucleus pulposus cells, n=3. **F.** Data for CLOCK are superimposed with corresponding sine waves fitted through CircWave analyses. **G.** MetaCycle-filtered circadian rhythmic gene expression in PER2 siRNA-transfected nucleus pulposus cells.

**Fig. S5. Hypoxia reversed the disruption of the circadian rhythm by HIF-1α. A.** Rhythmic expression of BMAL1, CLOCK, and LC3-II protein in hypoxia-treated circadian rhythm disrupted rat nucleus pulposus cells, n = 3. **B.** MetaCycle-filtered circadian rhythmic gene expression in the different conditions shown in panel A. **C.** Expression of HIF-1α protein in dexamethasone (Dex) and IL-1β-treated rat nucleus pulposus cells. **D.** Rhythmic protein expression of HIF-1α (Dex and Dex + IL-1β) as shown by MetaCycle analysis. **E.** MetaCycle-filtered circadian rhythmic gene expression of HIF-1α (Dex and Dex + IL-1β). **F.** CCK-8–based cell proliferation under normoxia vs hypoxia. Data are presented as mean ± SEM, n=3. Differences were analyzed by two-way ANOVA. ** P<0.01. **G.** Live/dead fluorescence imaging under normoxia vs hypoxia.

**Fig. S6. Effect of HIF1α and PER2 knockdown on the expression of BMAL1, CLOCK, PER2, and LC3-II in hypoxia-treated rat nucleus pulposus cells. A.** Western blot analysis showing protein expression of BMAL1, CLOCK, PER2, and LC3-II under different conditions: si NC, si HIF1α, and si PER2. **B.** MetaCycle-filtered circadian rhythmic gene expression for BMAL1, CLOCK, PER2, and LC3-II in HIF1α/PER2 siRNA-transfected rat nucleus pulposus cells.

**Fig. S7. Protein expression in rat nucleus pulposus cells treated with 5 μM KL001. A.** Expression of MMP-3, MMP-13, Collagen II, P62, LC3-II, and BMAL1 proteins following 5 μM KL001 treatment at the indicated time points (0, 6, 12, 24, 36, 48 hours). Data are presented as mean ± SEM, n=3. Differences were analyzed by two-way ANOVA. ** P<0.01. **B.** Quantification of MMP-3, MMP-13, Collagen II, P62, LC3-II, and BMAL1 protein expression compared with β-actin at different time points. Data are presented as mean ± SEM, n=3. Differences were analyzed by two-way ANOVA. ** P<0.01. **C.** Timeline of 5 μM KL001 treatment followed by medium change at the indicated time points. **D.** Protein expression of MMP-3, Aggrecan, and Collagen II after 5 μM KL001 treatment at the indicated time points (0, 6, 12, 24, 48 hours). **E.** Quantification of MMP-3, Aggrecan, and Collagen II protein expression compared with β-actin in D. Data are presented as mean ± SEM, n=3. Differences were analyzed by two-way ANOVA. ** P<0.01. **F.** Expression of MMP-3, MMP-13, Collagen II, Aggrecan in DEX, siPER2, IL-1β, and KL001 treated rat nucleus pulposus cells, as indicated. **G.** Quantification of MMP-3, MMP-13, Collagen II, and Aggrecan protein expression in F, n = 3, ** P<0.01, *** P<0.001.

**Fig. S8. Rabbit intervertebral disc degeneration model and measurement of oxygen content in rabbit intervertebral discs. A.** The vertebras and discs were exposed from the muscular space. **B.** The target discs were located under X-ray. **C.** The disc was punctured using a needle with a limiter. **D.** Successfully punctured disc. **E.** The disc was located under X-ray. **F.** The target disc was punctured using an oxygen content probe. **G.** Probe depth was adjusted under X-ray. **H, I.** In vitro example of the relationship between probe and nucleus pulposus.

Fig. S9. CUT&Tag profiling of HIF1α binding in rat nucleus pulposus cells under normoxia and hypoxia conditions. **A.** Genome-wide HIF1α binding profiles. Top: Average signal intensity plots centered on peaks (±2.0 kb). Bottom: Heatmaps of normalized read density across peaks, sorted by intensity (color scale: blue low to red high). Hypoxia shows enhanced binding compared to normoxia. **B.** IGV tracks at the PER2 locus, with increased HIF1α binding in hypoxia. **C.** Top 30 enriched GO terms for HIF1α-bound genes under hypoxia, ranked by -log10(p-value). Categories color-coded (black: biological process; gray: cellular component; purple: molecular function), including apoptosis regulation and signaling pathways. **D.** Bubble plot of top 30 KEGG pathways enriched in HIF1α-bound genes under hypoxia. Bubble size: gene count; color: -log10(p-value) (red: higher significance); pathways include non-small cell lung cancer and HIF-1 signaling.

**Fig. S10. Uncropped original Western B****lot images.**
